# Phytochemical Moieties From Indian Traditional Medicine for Targeting Dual Hotspots on SARS-CoV-2 Spike Protein: An Integrative *in-silico* Approach

**DOI:** 10.3389/fmed.2021.672629

**Published:** 2021-05-07

**Authors:** V. Umashankar, Sanjay H. Deshpande, Harsha V. Hegde, Ishwar Singh, Debprasad Chattopadhyay

**Affiliations:** ICMR-National Institute of Traditional Medicine, Indian Council of Medical Research, Department of Health Research (Government of India), Belagavi, India

**Keywords:** COVID-19, traditional medicine, docking, molecular dynamics, drug design

## Abstract

SARS-CoV-2 infection across the world has led to immense turbulence in the treatment modality, thus demanding a swift drug discovery process. Spike protein of SARS-CoV-2 binds to ACE2 receptor of human to initiate host invasion. Plethora of studies demonstrate the inhibition of Spike-ACE2 interactions to impair infection. The ancient Indian traditional medicine has been of great interest of Virologists worldwide to decipher potential antivirals. Hence, in this study, phytochemicals (1,952 compounds) from eight potential medicinal plants used in Indian traditional medicine were meticulously collated, based on their usage in respiratory disorders, along with immunomodulatory and anti-viral potential from contemporary literature. Further, these compounds were virtually screened against Receptor Binding Domain (RBD) of Spike protein. The potential compounds from each plant were prioritized based on the binding affinity, key hotspot interactions at ACE2 binding region and glycosylation sites. Finally, the potential hits in complex with spike protein were subjected to Molecular Dynamics simulation (450 ns), to infer the stability of complex formation. Among the compounds screened, Tellimagrandin-II (binding energy of −8.2 kcal/mol and binding free energy of −32.08 kcal/mol) from *Syzygium aromaticum* L. and O-Demethyl-demethoxy-curcumin (binding energy of −8.0 kcal/mol and binding free energy of −12.48 kcal/mol) from *Curcuma longa* L. were found to be highly potential due to their higher binding affinity and significant binding free energy (MM-PBSA), along with favorable ADMET properties and stable intermolecular interactions with hotspots (including the ASN343 glycosylation site). The proposed hits are highly promising, as these are resultant of stringent *in silico* checkpoints, traditionally used, and are documented through contemporary literature. Hence, could serve as promising leads for subsequent experimental validations.

**Graphical Abstract:**
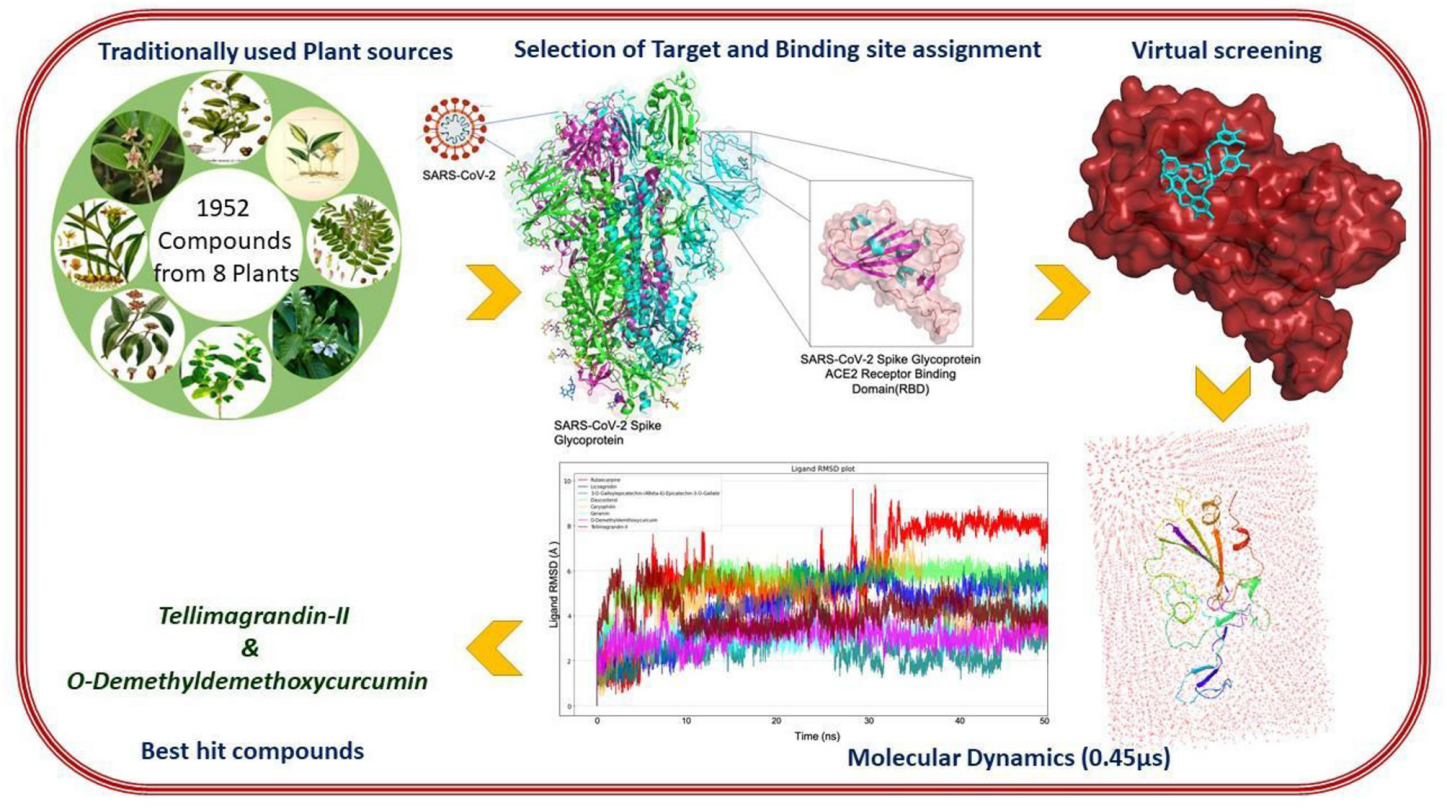
*In-silico* strategies implemented to prioritize phytochemical moieties for targeting Dual hotspots of Sars-Cov-2 spike protein.

## Introduction

**Figure 1:**
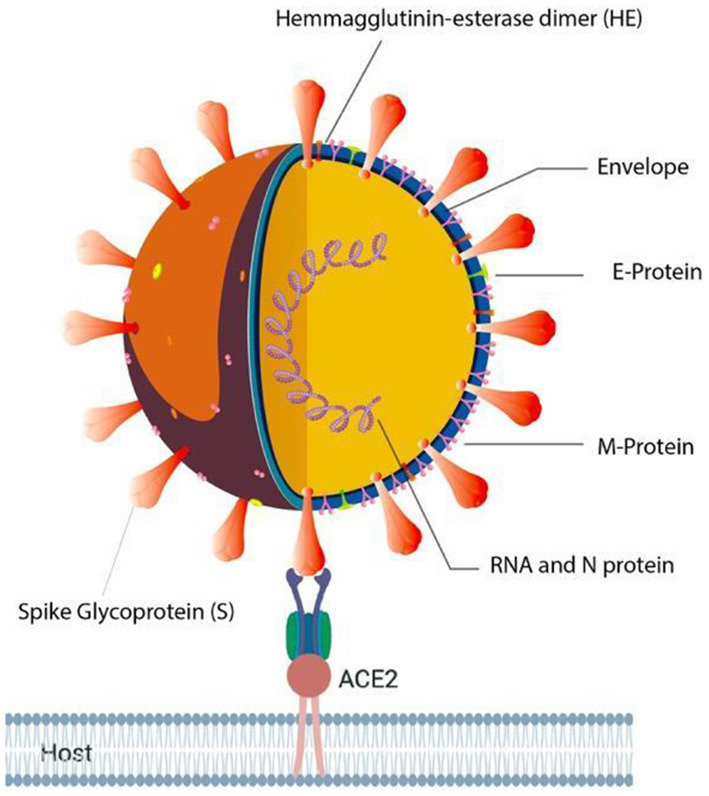
Illustration of SARS-CoV-2 in complex with Human ACE2 receptor.

A new respiratory infectious disease was reported in Wuhan, Hubei Province of China, around December 2019 (1, 2). The outbreak at the initial stage was linked to a seafood market with a possibility of animal transmission. In due course of time, human to human infection began that spread across the globe, and the disease was called as COVID-19 (Coronavirus disease 19). The newly emerged virus was named as SARS Corona virus-2 (SARS-CoV-2; Figure 1), based on the etiology and symptoms, which is closely associated with SARS-CoV identified in the year 2002 in China (3). The epidemic of COVID-19 has been declared as a pandemic by the WHO on 30th January 2020, which affected the population across the globe to the worst possible extent (4). The SARS-CoV-2 infection has spread across the continents, as of March 25, 2021 a total of 125,429,834 cases with a mortality of 2,756,742 and recoveries of 101,293,629 are reported, based on the registered cases (5). Currently, quarantine, isolation, use of masks, physical distancing, washing of hands with soap water and symptomatic treatment protocol is being strictly followed to manage the disease, as there is no drug available till date to selectively target this virus. These data mainly highlight the extent of spread across the globe. Hence, finding prophylactic or therapeutic agents becomes important and essential.

**Figure 2:**
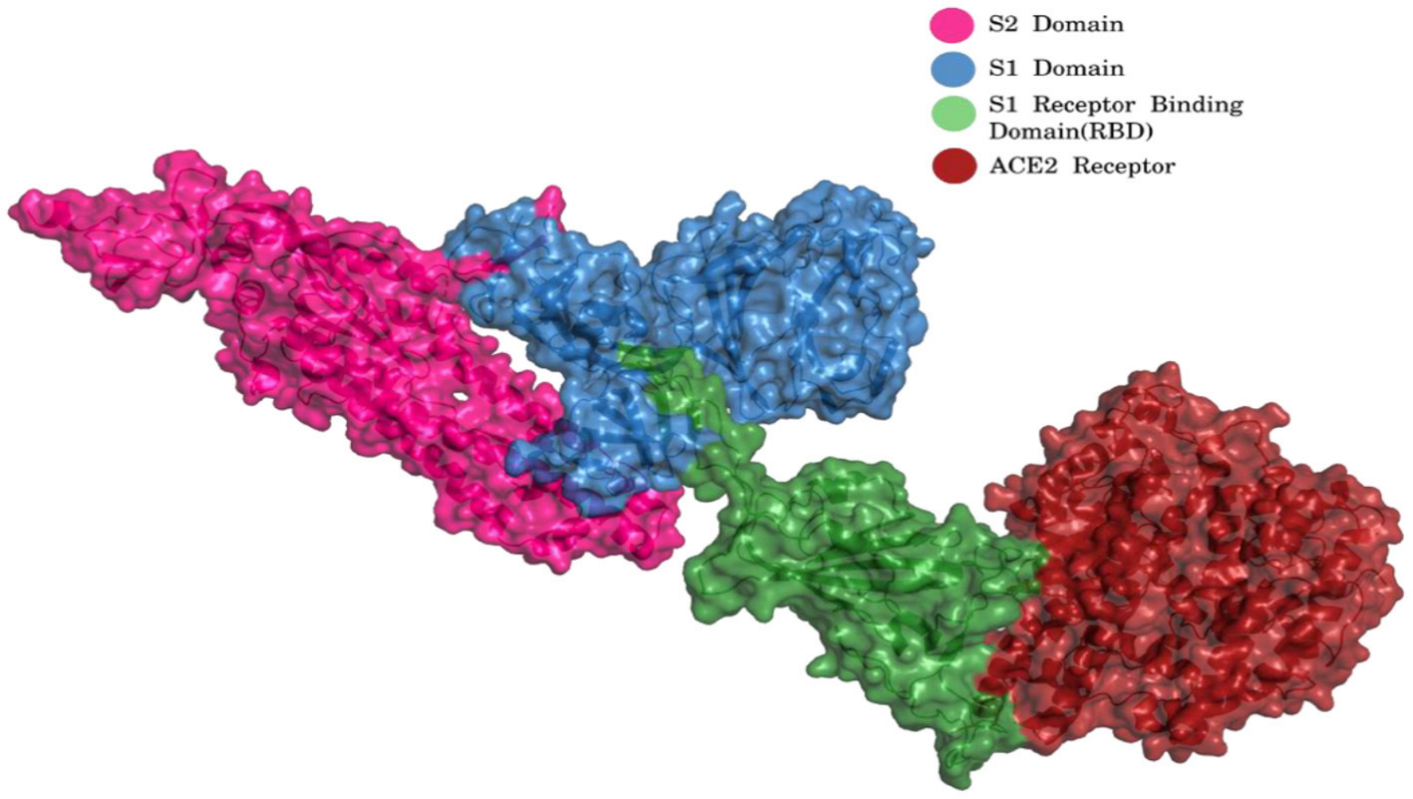
S1 and S2 region of spike glycoprotein and ACE2 receptor [Blue, S1 Domain; Pink, S2 Domain; Green, S1 Receptor binding domain (RBD); Red, ACE2 Receptor] (PDB ID 7KJ2).

The virus enters the human cells by the regulation of spike (S) glycoprotein (1,273 amino acids long; Figure 2) which is cleaved into 2 main units, namely, S1 (13–685 aa) and S2 (686–1,273 aa).

**Figure 3:**
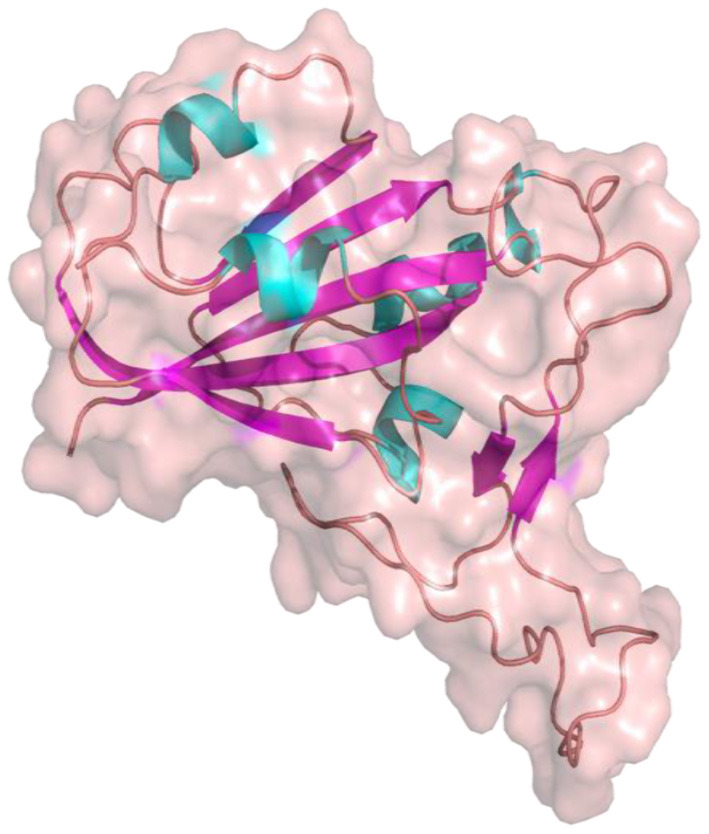
Receptor binding Domain of spike glycoprotein (Molecular Surface View) (PDB ID 7BZ5).

The S1 and S2 domains are present in individual monomers of the spike protein trimer (Supplementary Figure 1).This 3D structure of protein complex has been recently elucidated using Cryo-Electron microscopy (PDB id: 6VSB) (rendered using PyMol) (6, 7). The surface unit 1 (S1) helps in the strong attachment of the spike protein to human cell receptors. The cleavage of S1/S2 helps in the entry of viral particles and the fusion of the viral capsid with the host cell membrane is guided by the S2 subunit (8). Several studies established that angiotensin-converting enzyme 2 (ACE2) receptor of the host cell is the mediator that facilitates viral entry (9, 10). Spike protein S1 domain is further divided into multiple regions that are involved in binding to host receptors. In Spike protein, 319–541 aa region (S1) is known as receptor binding domain (RBD) [PDB id: 7BZ5; Figure 3; (11)]

and 437–508 as receptor binding motif (RBM), which binds to the ACE2 receptor. Other earlier studies have also compared the receptor-binding domain (RBD) of spike protein of both SARS-CoV and SARS-CoV-2 having high residue conservation, indicating that only a small change makes the SARS-CoV-2 binding to the ACE2 receptor different from the other coronaviruses (12, 13). Recent genome sequencing study also showed the spread of mutant form (D614G) of spike protein to have the potential for enhanced ACE2 binding (14). Glycosylation sites of SARS-CoV-2 are reported to modulate the host immune response, and also proposed to be the potential target for future mutations (15). The glycosylation process in coronaviruses mainly occurs to camouflage the immunogenic process in the host. Targeting glycosylation sites can also help in the early and rapid immune response to neutralize the virion (16). The potential hotspot residues of spike protein namely, THR323, SER325, ASN331, and ASN343 are reported to be involved in glycosylation. Among these residues, ASN343 spans the RBD region of the spike protein (17). Thus, targeting these sites could be an efficient mode for combating SARS-CoV2 infections.

Drug development is a tedious and highly time-consuming process usually takes years to get the newly developed drug for the treatment (18–20). Thus, one of the most preferred method is to find a suitable drug through drug repurposing, as it saves both time and financial resources (21). The most common source of drug repurposing is existing drugs or molecules of natural origin, and the scientists across the globe feel that the use of compounds from natural sources is one of the best and sought about way to move forward (22), even to find a drug for COVID-19 (23, 24). Computational approaches in identifying potential hits have gained momentum due to their cost effective and time saving efficiencies in drug discovery process (25). Moreover, these approaches have been well-utilized for mining potential chemical moieties from diverse phytochemical libraries (24, 26, 27). In recent times, studies on traditional medicine have climbed to newer heights across the globe due to its immense potential, easy availability, time-tested safety profile, and wide range of pharmacological actions. The increase in studies is also due to the implementation of technologies to understand the structure and function of phytocompounds from nature (28). With the continuous advancement in the field of computer science, many drugs have been approved from natural sources through computer-aided drug design, like Ponatinib (FDA approval: 2012), Dasatinib (FDA approval: 2006), and Imatinib (FDA approval: 2001). Applications of *in silico* approach helps to calculate and analyze the combinations of compounds and targets as highly accurate, hence gaining more and more importance in the field of drug discovery by saving time and money (29). Several studies revealed that ethnomedicinal “phytophores” belonging to different classes, based on their structure activity relationship (SAR), showed effectiveness in curtailing viral replication in diverse viral infections. The antiviral compounds usually interfere in host-virus interaction points like viral entry process from adhesion/attachment to fusion and penetration, inhibit enzymatic activity, and or block one or more steps of the viral life cycle including replication to release. The scientific evidence and the traditional usage of antiviral plant extracts clearly portray the potential of natural compounds in modulating viral infection (30).

India is a country with rich biodiversity and long history of use of traditional medicine (TM) with a vast knowledge base of useful medicinal plants through the ages. Indian Ayurveda is one of the oldest systems of medicine of the world existing since the world's first civilizations and Vedic era. The main resource of TM is the generation-old time-tested knowledge base of plant-based formulations and wisdom of different communities, known as “ethnomedicine” (31), using different parts of plants from roots to leaves, bark fruits, and seeds (*New Look to Phytomedicine*, 2019). A wide range of plants used in ethnomedicinal practices were shown to be highly effective in the management of diverse viral infections by inhibiting either the viral life cycle or the host-virus interactions (32).

Viral infections are increasing across the globe mainly due to increased anthropogenic activities like land-use change, increased human-animal interaction and lack of proper healthcare infrastructure. Hence, the discovery of antivirals from natural sources, mainly traditionally used medicinal plants have gained importance. Since ages, plants have been used as a source of therapeutics in diverse ethnomedicinal practices. Many ethnomedicinal plant extracts and phytocompounds are known to modulate host immune responses (33) and may exert antimicrobial and antiviral effects (34). A variety of plant compounds including alkaloids, coumarins, essential oils, flavonoids, polyphenols, phytosterols, proteins, peptides, saponins, and tannins play diverse roles in the human system. Consistent progress has been made in the development of nature-based antiviral drugs in recent years, as natural products like plant extracts and phytocompounds used in TM are novel and broad-based chemical entity that may serve as a potential sources of antiviral drugs (35). The ever-increasing drug resistance, frequent microbial mutations with increased emerging and re-emerging outbreaks of viruses necessitate the development of easily available cost-effective antimicrobials and antivirals for better treatments. Hence, traditional medicines are the hope and source for novel agents to manage viral diseases (30). A whole range of viral diseases caused by the Dengue, Human herpes viruses, HIV, Rabies, and Severe acute respiratory syndrome (SARS) needs potential therapeutics; while using modern tools the vast knowledge of ethnomedicinal practices can be identified and validated for antiviral applications (36). Thus, a surge of research is been observed in research institutes and universities, particularly the countries rich in TM.

In recent times, several phytocompounds having antiviral potential have been identified with their molecular mechanism of action. Spiroketalenol, isolated from the rhizome extract of *Tanacetum vulgare* L., was found to inhibit HSV-1 and HSV-2 by blocking the virus entry, and inhibit the activity of viral glycoproteins (37). Another compound Samarangenin B from the roots of *Limonium sinense* found to suppress the replication of HSV-1 by inhibiting the expression of HSV-1 immediate early (IE) or α- gene (38). Harmaline (HM), a dihydro-pyrido-indole, from the ethnomedicinal herb *Ophiorrhiza nicobarica* is reported to exhibit anti-HSV activity by suppressing the viral IE gene synthesis through epigenetic blocking of LSD-1 with a different mode of action than the gold standard antiviral Acyclovir (39). Further, it was reported that the ursolic acid isolated from *Mallotus peltatus* (Geist) Muell. Arg. dose-dependently inhibits the plaque formation of both HSV-1 and HSV-2 at 10 μg/ml within 2–5 h post-infection (40). Moreover, *Odina wodier* Roxb, a herb used in folklore medicine confer therapeutic effects on the skin infections caused by HSV (41). *Pterocarya stenoptera* traditionally used in the treatment of viral diseases is another potential plant with antiviral activity and its isolated compound Pterocarnin A was shown to inhibit HSV-2, by blocking the penetration of the virion into the host cells (42). Complementarily, many bio-active compounds from plants were shown to have immunomodulatory activities by triggering anti-inflammatory responses, which in turn helps in the control of viral infection (34). Earlier studies have revealed that modulation of NF-κB signaling mediated anti-inflammatory response triggered by *Pedilanthus tithymaloides* L. confer a higher level of anti-HSV activity (43, 44). Further, ultrasound-induced Gallic acid based gold nanoparticles can inhibit HSV infection with EC_50_ of 32.3 and 38.6 μM against HSV-1 and HSV-2, respectively (45). While oleo-gum resin-extract and β-Boswellic acid of *Boswellia serrata* inhibit HSV-1 infection through modulation of NF-kβ and p38 MAP kinase signaling (46).

Ethnomedicinal literature claims the broad-spectrum antiviral activity of diverse medicinal plant extracts and phytocompounds, as the majority of those antiviral herbs contain flavones, polyphenols, and alkaloids. Due to the rapid emergence of new highly infectious viruses as well as re-emergence of drug-resistance, and difficult-to-treat infections along with the concurrent availability of advanced technological tools, the exploration of antiviral activity of medicinal plants has acquired momentum. In the current scenario of COVID-19, traditional Chinese medicine (TCM) was included in the guideline for the treatment, which claimed to be efficacious in several cases (47). Similarly, many of the Indian ethnomedicinal plants are reported to ameliorate the symptoms related to COVID-19, with antiviral activities (24). The hits based on such observations can provide the edge for development of drugs to manage/treat COVID-19. Thus, in this study we have performed a meticulous analysis of documented antiviral properties of selected traditionally used Indian medicinal plants. This resulted in eight potential plants to be probed for phytochemical moieties that could target COVID-19 effectively. The rationale on selection of plants is discussed in detail as follows.

## Materials and Methods

### Selection of Plants and the Rationale

#### *Tylophora indica* Burm F. Merrill (Asclepiadaceae) Syn. *T. asthmatica* (Roxb) Wt & Arn.

**Figure 4:**
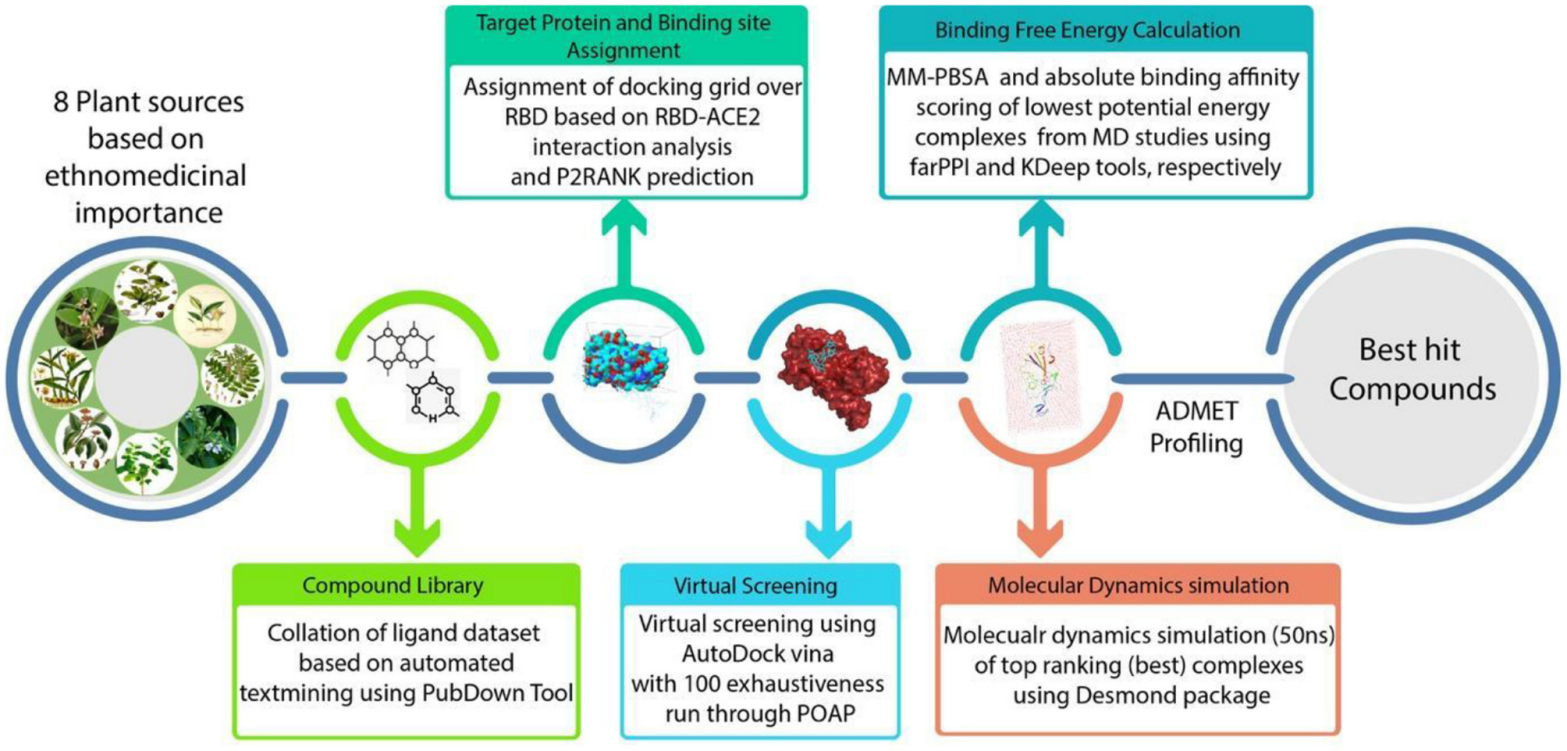
A schematic representation of methodologies implemented in this study.

The detailed flowchart on the insilico methodologies implemented in this study towards prioritization of phytochemical moieties are shown in Figure 4. *Tylophora indica*, a perennial climber indigenous to India, commonly called “Antamool” is an important medicinal plant used in Indian medicine, mainly found in the plains, hills, and the forest borders in eastern and southern India. This plant is ethnically used for treating various types of ailments including cancer, respiratory infections, bronchial asthma, whooping cough, and anaphylaxis. The active ingredients of *T. indica* are mainly available in leaves and roots that exhibit most therapeutic effects mostly due to the pharmacologically active alkaloids tylophorine, tylophorinine, and tylophorinidine (48). Some previous studies have shown that Tylophora alkaloids can inhibit viral protease and suppresses viral RNA replication by blocking the JAK2 mediated NF-κB activation (49). While tylophorine derivatives have inhibitory effects on mouse hepatitis virus (MHV), transmissible gastroenteritis virus (TGEV), and SARS-CoV (50–52). A recent report revealed that Tylophora alkaloids could inhibit the CoV-infected cells of swine (53). However, an earlier pharmacokinetic study demonstrated moderate to good oral bioavailability of tylophorine (65.7%) in rats (50). Recent studies also showed that alkaloids from *T. indica* possess anti-replication activity and inhibit the cytopathic effect induced by apoptosis, and apoptosis induced by viral infection (54). While kaempferol derived from *T. indica* could effectively block the 3a channel protein in coronavirus (55).

#### *Glycyrrhiza glabra* L. (Fabaceae)

*Glycyrrhiza glabra* rhizome (Yashtimadhu) is used worldwide in various traditional systems of medicines. In Ayurveda it is an important drug component of Dasamoolarishtam, Aswagandharishtam, Madhu-yastyaditaila etc., as mentioned in Charaka Samhita. In folk medicine it is used as a laxative, emmenagogue, contraceptive, galactagogue, anti-asthmatic, anti-tussive, and antiviral agent. Being a member of the pea and bean family, the plant is best known for its use in making liquorice-flavored confectionery while roots and rhizomes are used for medicinal purposes. A number of pharmacological effects including expectorant and antitussive, antiviral against SARS-CoV, HIV, and in the treatment of diabetes, cancer, and hepatitis (56) have been studied for this plant. The main chemical constituent of liquorice is glycyrrhizin, a triterpene saponin with a low haemolytic index; while the root contains glycyrrhetinic (Glycyrrhetic) acid, the aglycone of glycyrrhizin. Other active constituents of liquorice include isoflavonoids, chalcones, coumarins, triterpenoids, sterols, lignans, amino acids, amines, gums, and volatile oils (57). Chemically *G. glabra* comprises of 20 triterpenoids and around 300 flavonoid compounds. Among these 18β-glycyrrhetinic acid, glycyrrhizin, glabridin, licochalcone A, licochalcone E, and liquiritigenin have antimicrobial activity (58). While glycyrrhizin A and 18β-glycyrrhetinic acid can elicit anti-HCV activity through inhibition of core protein expression and by blocking the degradation of NFκB inhibitor IκB, followed by activation of T lymphocyte proliferation (58). The glycyrrhizin and its analogs have significant inhibitory effect against hepatitis, herpes, influenza, and SARS viruses (59). Oral administration of *G. glabra* extract has an antitussive effect by promoting pharyngeal and bronchial secretions leading to good expectorant action. Liquiritigenin, a flavonoid from the root extracts demonstrated anti-asthmatic activity (60). The antiviral activity of glycyrrhizin have been assessed against two clinical isolates of coronavirus (FFM-1 and FFM-2) from SARS patients and found that it could inhibit viral adsorption, penetration, and replication (59). The crude Glycyrrhizin was also demonstrated to have low antiviral activity against varicella zoster virus (VZV) better than acyclovir and interferon (61). The roots of *G. glabra* had an accumulation of molecules having 3D similarities to influenza neuraminidase (NA) inhibitors. Further, it was elaborated in chemiluminescence (CL)-based NA inhibition assays on different influenza virus strains including an oseltamivir-resistant isolate A/342/09 (H1N1) that 11 out of 12 compounds had IC_50_ in nanomolar to micromolar range (62). A study with *G. glabra* leaf extract also revealed antiviral activity against Newcastle disease virus (NDV) with an highest embryo survival rate at 300 μg/ml (63).

#### *Camellia sinensis* L. (Theaceae)

The use of *Camellia sinensis* or tea as beverage and medicine has a long history of almost 5000 years. Chemically tea contains polyphenols, flavonoids, tannins, and caffeine derivatives with amino acids, having antioxidant and diverse therapeutic effects. Black tea is prepared from the green tea leaves by a series of fermentation when catechin (30%) of green leaves oxidized into theaflavins (theaflavin, theaflavin-3-gallate, theaflavin-3′-gallate, and theaflavin-3,3′-digallate) by dimerization and into thearubigins (17%) through polymerization. Tea flavonoids help in the reduction of inflammation, possess antimicrobial effects, and are used in the treatment of respiratory diseases such as asthma. A number of compounds like theaflavins and tannins from black and green tea have antiviral activities, mainly against bovine rotavirus and bovine coronavirus (32, 64). *In vitro* studies have shown that theaflavin di-gallate inhibited the infectivity of influenza A and B viruses (65). Green tea is widely used as a beverage across the world, mainly for its antioxidant nature. It is rich in polyphenolic compounds (flavonoids) and bonded benzene rings combined with multiple hydroxyl functional groups. A study on water-soluble phenols like tannic acid and theflavin-3-3′-digallate have shown to inhibit 3-chymotrypsin like protease (3CLpro) of SARS Coronavirus. Hence, it can be considered as a starting point for molecules against the SARS-CoV-2 (32, 66). A recent docking study revealed that the bioactive molecules of *C. sinensis*: Barrigenol, Kaempferol, and Myricetin have significant binding affinity with the active site of SARS-CoV2 Nsp15 protein (67). In a similar study, Oolonghomobisflavan-A, Theasinensin-D, and Theaflavin-3-O-gallate from tea were compared with repurposed antivirals (Atazanavir, Darunavir, and Lopinavir) for their binding affinity with Mpro of SARS-CoV-2. The results revealed that Oolonghomobisflavan-A to be highly significant in terms of binding affinity and intermolecular interactions when compared to all the other repurposed antiviral inhibitors (68).

#### *Justicia adhatoda* L. (Acanthaceae) Syn. *Adhatoda vasica* Nees

*Justicia adhatoda* (synonym *Adhatoda vasica*), known as Vasaka in Ayurveda, is a well-known medicinal plant in indigenous system of medicine, mostly effective in treating respiratory ailments, as the leaf extract has a stimulant effect. Vasica leaf is an antispasmodic cum expectorant and has been used for centuries to treat asthma, chronic bronchitis, and other problems including fever, swelling, asthma, pneumonia, malaria, tuberculosis, cough, and cold (69). The infusion of *A. Vasica* leaf is known to relieve headaches. The root is used as an expectorant and antispasmodic; while the root infusion has an anthelmintic property. The phytochemical profiling of this plant showed the presence of alkaloids, anthraquinones, flavonoids, phenols, saponins, and tannins (70). Several studies showed that the aqueous and methanolic extracts of leaves can directly interfere with the envelop proteins of many viruses. In particular, methanolic extract had a higher level of inhibition of influenza virus, by blocking viral attachment and inhibition of viral hemagglutinin (HA) protein. Detailed study revealed that the methanolic extract mainly comprised of Vasicine alkaloids have antiviral activity (71). Aqueous extract of leaves is reported to inhibit the arachidonic acid metabolites through COX (TXB2) and LOX (LP1 and 12-HETE) pathways; while platelet aggregation studies showed butanol extract to exert strong inhibition against arachidonic acid, platelet activating factor, and collagen-induced aggregation (72). Methanolic extract also possess antiviral activity against HSV-2, while aqueous extract against HSV-1. Moreover, the methanolic extract showed 100% reduction in HA at 10 mg/ml; while the aqueous extracts at 5–10 mg/ml dose reduced the HA levels to 33 and 16.67%, respectively, suggesting strong anti-influenza activity by inhibiting viral attachment and/or replication (73).

#### *Ocimum Tenuiflorum* L. (Lamiaceae) Syn. *O. sanctum* L. (Tulsi)

*Ocimum sanctum* or Tulsi has been used for thousands of years for its diverse therapeutic activities, and is known as the “Queen of herbs” or the legendary “Incomparable one” of India with strong aroma and astringent taste. It is the holiest and most cherished plant for its healing and health-promoting properties and in TM, Tulsi is known as an adaptogen that balances different processes in the body and helps in adapting stress. Ayurveda treats it as a kind of “elixir of life” and is believed to promote longevity and a healthy body. Thus, extracts from Tulsi are used in many Ayurvedic remedies including the common cold, headache, stomach ailments, inflammation and heart disease (74). Several studies with *O. sanctum* leaf extracts showed therapeutic, prophylactic, and virucidal activities. A study *in ovo* model indicated its therapeutic activity against H9N2 virus by reducing the infection level (75); while crude extracts or individual compounds isolated from Tulsi have a wide spectrum of antiviral activity against HSV, Adenovirus, Coxsackievirus B1, and Enteroviruses (76). Tulsi is used in diverse formulations including mouthwash, sanitizer and water purifiers (77). The purified components apigenin, linalool, and ursolic acid of *O. basilicum* showed a broad spectrum of antiviral activities against DNA viruses (HSV, adenoviruses, hepatitis B virus) and RNA viruses (Coxsackievirus B1, Enterovirus 71), among which ursolic acid showed the strongest activity against HSV (40), ADV-8, CVB1, and EV71 (76). Crude, terpenoid, and polyphenol-rich extract of *O. sanctum* showed significant virucidal activity (*p* < 0.001–0.01) and was found to decrease the virus genome copy numbers at the lowest dose up to 72 h post-infection (77). Recently, molecular docking studies also suggest that tulsinol A-G and dihydro-dieuginol B as potential inhibitors of SARS Coronavirus Main Protease (Mpro) and Papain-like Protease (PLpro), indicating that *O. sanctum* can be used as preventive against CoV due to its potential immunomodulatory, ACE2 blocking and viral replication inhibition properties (78).

#### *Zingiber Officinale* Roscoe (Zingiberaceae)

*Zingiber officinale* (Ginger), native to South-East Asia, is used as a common spice across the world. It encompasses several diverse chemical moieties with antiarthritic, anti-inflammatory, antidiabetic, antibacterial, antifungal, and anticancer activities and is one of the major medicinal sources of Ayurveda, Unani, Siddha, and various traditional medicine systems of India (79). Fresh ginger is used to treat cold, nausea, colic, heart palpitations, respiratory illnesses, dyspepsia and dry cough. During the nineteenth century a popular formulation from Ginger was used in the treatment of asthma and cough, consisted of the mixture of fresh ginger, and fresh garlic juice with honey (80). The ginger rhizome contains highly pungent vanillyl ketones like Gingerol and paradol derivatives having therapeutic effect on a wide range of diseases (81). Fresh rhizomes have inhibitory activity against the human respiratory syncytial virus (RSV) that infects the respiratory tract of humans (82). It was shown that the water-grown ginger has greater inhibitory activity against Chikungunya (CHIK) virus (83). The ginger oil was also reported to inhibit HSV-2 plaque formation (84) while the dried rhizomes containing sesquiterpenes have anti-rhinoviral activity in plaque reduction assay, but the best activity was found with the beta-sesquiphellandrene at an IC_50_ of 0.44 μM *in vitro* (85).

#### *Curcuma longa* L. (Zingiberaceae)

*Curcuma longa* (Turmeric) belongs to the ginger family Zingiberaceae; and turmeric rhizome has been traditionally used in India for various ailments and diseases. Indian traditional and folklore medicine used turmeric to treat inflammation, infections, respiratory illness, gastric, hepatic, and blood disorders. Curcumin, the marker compound of turmeric is a well-studied therapeutic phyto-molecule, while curcumin and its derivatives are the major polyphenols of the rhizome (86). The antiviral activity of curcumin and its derivatives have been established against a wide variety of pathogenic viruses including hepatitis, herpes simplex, human immune deficiency, human papilloma, influenza, and zika. The mechanism is mainly by inhibition of viral entry, replication, particle production, viral protease, and gene expression (87). Curcumin and its analogs also modulate the regulation of renin-angiotensin–aldosterone system (RAAS) which is involved in anti-inflammatory, anti-oxidant and anti-hypertensive activity, that are highly elevated in viral infection (88). Crude aqueous and ethanolic extracts of *C. longa* confer significant antiviral activity against H5N1 virus *in vitro* by inhibiting viral replication with significant upregulation of TNF-α and IFN-β mRNA expressions (89). Anti-influenza activity of curcumin was earlier assessed by computational methods, wherein curcumin derivatives were docked against the HA protein of influenza (H1N1) virus. The results inferred that specific curcumin derivatives can be successfully used against influenza virus infection. Moreover, curcuminoids from the methanol extract of *C. longa* also provide strong inhibitory effects on the neuraminidases of H1N1 and H9N2, as non-competitive inhibitors (90).

#### *Syzygium Aromaticum* (L.) Merr. & L. M. Perry (Myrtaceae)

Clove, the aromatic flower bud of *Syzygium aromaticum* is one of the ancient traditionally used spices in almost every household in India, with several therapeutic properties for dental, digestive, and respiratory disorders, including asthma (91). The other application of clove includes food preservation. Cloves contain various classes of phytochemicals including sesquiterpenes, monoterpenes, hydrocarbons, and phenolics along with Eugenyl acetate, eugenol, and β-caryophyllene as principal components of clove oil. Various pharmacological studies with clove have shown its inhibitory effects on pathogenic bacteria, *Plasmodium*, and Herpes simplex and Hepatitis C viruses (92). The essential oil of clove contains 85–95% eugenol and is shown to be highly effective in the treatment of HSV and HCV by blocking viral replication. The synergistic action of acyclovir and *S. aromaticum* extract have a significant impact on the inhibition of viral replication (92). Aqueous extract of clove showed antiviral activity against Feline Calicivirus (FCV) as a surrogate for human norovirus. Pre-treatment of FCV with clove oil reduced viral titer to 6.0 logs. The antiviral activity of the pure eugenol was similar to the clove extract, albeit at a lower level (93). The silver nanoparticles prepared from the aqueous extract of the flower buds of *S. aromaticum* were found to be novel and effective against the Newcastle Viral Disease (NDV) *in vitro* and in embryonated eggs (94).

### Data Sources

In this study, the dataset of phytochemicals of eight plants were acquired from different sources like CMAUP (95), NPASS (96), Dr. Dukes Database and KnapSack (97) database (Supplementary Table 1). Initially, the list of all the phytochemicals were collated out from individual sources by manual curation. In the next step, all the duplicate entries and the ubiquitous chemicals were removed from the list. The final list was taken as input for downloading structural files from the PubChem database using an automation script created using Python programming (https://github.com/sandes89/PubDown). The structures which were accessible online and documented were only considered for screening.

### Docking Studies

#### Preparation of Protein

The crystal structure of RBD (PDB id: 7BZ5) of spike protein presented in Figure 3 was downloaded from RCSB PDB (Protein Data Bank) (98, 99). GUI based “Auto-Dock Tools” was used to prepare and execute the docking studies. Kollman atom charges, solvation parameters, and polar hydrogens were added to the protein and proceeded for docking studies. As the ligands used are not peptides, Gasteiger charges were assigned only to the protein and the non-polar hydrogens were merged. Based on the literature and predicted active regions, a grid box was assigned around the active sites using AutoGrid application (100).

#### Preparation of Ligands

The 2D/3D structures were retrieved from PubChem Database using a custom written python script which is hosted on GitHub portal (https://github.com/sandes89/PubDown). List of compounds with their chemical names were prepared as an input to Python script and searched iteratively on PubChem ftp database and the compounds were downloaded in sdf (Structure Data File) format. A total of 1,952 compounds were downloaded from PubChem database. In this study, POAP (101) was used for the preparation of ligands and for virtual screening. POAP tool is a bash shell script-based pipeline which can be used to optimize ligands for docking using Open Babel (102) and to perform virtual screening using Autodock Suite. POAP implements dynamic file handling methods for efficient memory usage and data organization, ligand minimization (5,000 steps), MMFF94 force-field was employed with the addition of hydrogens. A total of 50 conformations for each compound were generated using the weighted rotor search method, with minimization using the steepest descent method. Finally, the best conformation was retained in.pdbqt format for utilization in further docking studies.

#### Active Residues Definition and Cavity Prioritization

The most important aspect in docking studies is the identification of important residues and favorable cavity for ligand binding. In this study, the cavity definition was mainly performed based on the literature insights on important residues (ACE2 binding site) coupled with the cavity prediction using P2RANK (103). The P2RANK predicted cavity spanned the ACE2 binding residues, as well as on few glycosylation sites. It should be noted that viral glycosylation has many roles in viral pathogenesis and biology, as it affects protein folding and stable interaction with host cells (15). As discussed earlier, glycosylation process in coronaviruses mainly occur to camouflage the immunogenic process in the host. Targeting glycosylation sites can aid in the primary and rapid immune response to neutralize the virus (16). Hence, along with ACE2 binding residues, glycosylation sites spanning the active cavity predicted by P2RANK were also considered for grid box generation. Considering the importance of the binding site with ACE2, glycosylation sites as well as the active cavity predicted by the P2RANK tool, the docking grid for molecular docking was fixed.

#### Virtual Screening Using POAP and ADMET Prediction

The geometry optimized compounds were subjected to Molecular docking with SARS-CoV-2 Spike glycoprotein. In the docking process, the ligands were considered as flexible and protein was considered as rigid body. The Grid box was prepared based on the active site residues as inferred from earlier ligand co-crystallized complex of spike protein and P2RANK based binding pocket prediction. For the docking process, an exhaustiveness value of 100 was fixed in Vina. The resulting Protein-ligand complexes were analyzed for intermolecular interactions using PLIP tool (104). The top-ranking ligands were subjected to ADMET profiling using pKCSM server (105).

#### Molecular Dynamics (MD) Simulation

The MD simulation of Apo protein and docked complexes were carried out using Desmond version 2020. Here, OPLS_2005 force field was used to initiate the MD simulation, and the system was solvated using SPC (Simple point charge) water model (106). The neutralization of the system was performed by adding counter ions and the details of ions and concentration added to complexes are given in Supplementary Table 11. Energy minimization of the entire system was performed using OPLS_2005, as it is an all-atom type force field (107). In the studies on natural compounds, the application of OPLS_2005 force field was found to be highly optimal. Hence, it was adopted for this study (27). The geometry of water molecules, the bond lengths and the bond angles of heavy atoms was restrained using the SHAKE algorithm (108). Simulation of the continuous system was executed by applying periodic boundary conditions (109) and long-range electrostatics was maintained by the particle mesh Ewald method (110, 111). The equilibration of the system was done using NPT ensemble with temperature at 300 k and pressure at 1.0 bar. The coupling of temperature-pressure parameters was done using the Berendsen coupling algorithm (112). On post-minimization and equilibration of the system, the Apo protein system consisted of 28,645 atoms in total and number of atoms for all the complexes are given in Supplementary Table 11. On post-preparation of the system, the production run was performed for 50 ns with a time step of 1.2 fs and trajectory recording was done for every 5.0 ps summing up to the recording of 10,000 frames. The calculation of the RMSD (Root mean square deviation) was done for the backbone atoms and was analyzed graphically to understand the nature of protein-ligand interactions (113, 114). RMSF (Root Mean Square Fluctuation) for every residue was calculated to understand the major conformational changes in the residues in comparison between the initial state and dynamics state (115). The compactness of the protein-ligand complexes in comparison to Apo form was calculated using radius of gyration rGyr (116). The 2D interactions of Protein-ligand complex showing the stability of the complexes and interaction sites were generated for the complete run time.

#### KDeep Based Absolute Binding Affinity (ΔG) Calculation

On post-molecular dynamics simulation, the top ranking complexes from each plant were energy minimized and was also analyzed for absolute binding affinity (ΔG) using KDeep (117). KDeep employs machine learning approach with implementation of 3D convolutional neural networks. KDeep analyses the input and voxelizes into pharmacophore features like (aromatic, hydrophobic, hydrogen-bond acceptor/donor, positive, and negative ionizable). The prepared input is passed to the DCNN (Deep Convolutional Neural Network) model which is pre-trained by PDB bind v.2016 database, wherein, based on adaptability to the model the absolute free energy of the protein-ligand complex is calculated.

#### MM-PBSA Calculation of Topmost Stable Complexes

Molecular Mechanics-Poisson Boltzmann Surface Area (MM-PBSA) calculation is one of the most commonly used method for enumerating binding free energy of protein-ligand complexes. The MM-PBSA combines energy calculations based on molecular mechanics and implicit solvent model. This method precisely estimates the binding free energy of the protein-ligand complex, which is estimated by the differences between the free energy of the complex and free energies of unbound individual components of the complex (118). In this study, the MM-PBSA (PB3) based binding free energy of top most stable complexes were calculated using farPPI server. WhilePB3 option was considered as it was benchmarked to be highly accurate when compared to all the other methods in FarPPI (119). The force fields, GAFF2 and ff14SB as provided in the server were applied for ligand and protein, respectively. This calculation was performed for the lowest potential energy conformation of the top most stable complexes.

## Results

### Binding Site Assignment

**Figure 5:**
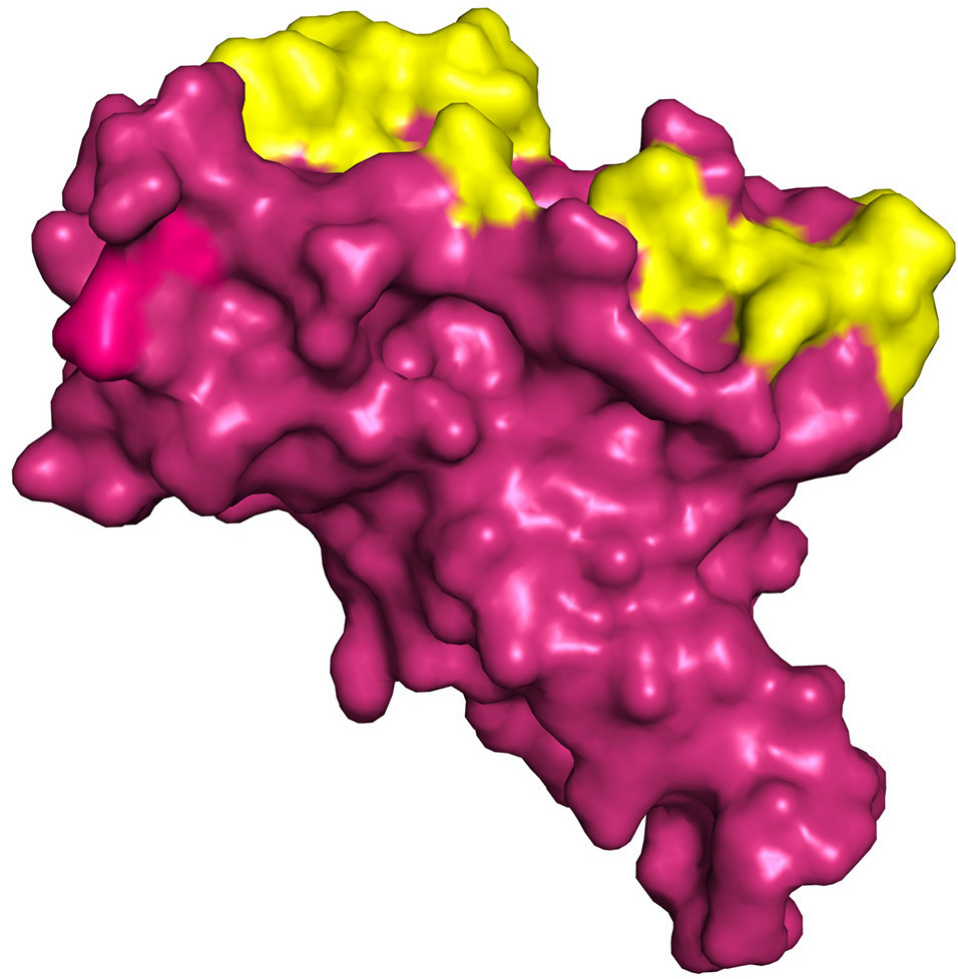
Represents the region of docking grid fixation (yellow surface) based on the documented active site residues and P2RANK prediction including glycosylation site (PDB ID 7BZ5).

The structure of spike protein co-crystallized with the ligand is yet to be available in PDB, hence P2RANK was used to identify the potential drug binding pockets in concurrence with key hotspot residues reported in the literature. Based on the interaction plot of Spike protein (RBD)-ACE2 receptor complex (PDB ID: 6M0J), the residues of Spike protein that are involved in the interactions were identified as GLY502, THR500, LYS417, TYR449, GLY446, ASN487, and GLY496. In addition, the P2RANK prediction also covered the ASN343 glycosylation site spanning the RBD of spike glycoprotein. Considering all these important residues based on the literature, cavities and solvent accessible areas as predicted by P2RANK, an optimal docking grid box was created over the spike protein (PBD ID: 7BZ5). It should be noted that the 6M0J structure was only utilized for mapping the ACE2-Binding region on the spike protein, and was not used for docking studies, as some atoms were found missing in the structure. Hence, the 7BZ5 structure (ACE2 unbound form) with no such issues were utilized for docking and simulation studies. The Grid file was generated with the following coordinates (*x* = −85.56, *y* = −23.44, *z* = −16.90) using Autodock tools program and was proceeded for molecular docking using Vina as shown in Figure 5.

### Molecular Docking Studies

In pursuit of finding an important candidate for managing COVID-19 from selected plants, molecular docking studies were carried out with phytochemicals listed from eight plants on the binding pocket of COVID-19 spike glycoprotein (PDB ID: 7BZ5). Based on literature review, it was clearly found that the virus enters the human cell via the ACE2 receptor. Hence, we prioritized the receptor binding domain of spike glycoprotein PDB ID: 7BZ5, wherein the spike protein interacts with ACE2 for docking studies. The geometry optimized compounds from all the plants were docked against active-cavity as discussed above, and were ranked based on their corresponding docking score. Compounds having the docking score of < -7.0 kcal/mol were considered for further evaluation. This cut-off was adopted, based on earlier studies, wherein it was found to be optimal (120, 121). A comprehensive evaluation of all the compounds was performed based on the binding affinity score and the involvement of key residues in the binding cavity (Table 1). Also the ADMET profiling of the compounds with topmost binding affinity was carried out using pKCSM server (105) and the data is provided in Supplementary Table 10.

**Table 1:** Compounds from each plant with best binding energy/score.

**Plant id**	**Plant name**	**Compound name**	**Binding energy**	**KDeep ΔG (Kcal/mol)**	**Interactions**		
			**(kcal/mol)**		**Hydrogen bond (Distance in Å)**	**Hydrophobic interaction (Distance in Å)**	**Pi-stacking**
NITM1	*Tylophora indica* (Burm. F.) Merrill Syn. *Tylophora asthmatica* (Roxb.) Wt & Arn.	Rutaecarpine	−7.9	−6.15	SER371 (3.23), SER373 (3.14)	PHE342 (3.16), VAL367 (3.39), LEU368 (3.67), PHE374 (3.64)	TRP436
NITM2	*Glycyrrhiza glabra* L.	Licoagrodin	−8.7	−9.45	GLY339 (3.13), ASP364 (3.49), VAL367 (4.06), SER371 (3.13)	PHE338 (3.89), GLU340 (3.71), ASP364 (2.93), VAL367 (3.69), LEU368 (3.46), PHE374 (3.93), TRP436 (3.83)	
NITM3	*Camellia sinensis* L.	3-O-Galloylepicatechin-(4Beta-6)-Epicatechin-3-O-Gallate	−8.3	−10.95	PHE338 (2.46), ASN370 (2.17), SER71 (2.57), SER373 (2.21), ASN437 (3.44), ASN440 (2.67)	PHE338 (3.77), PHE342 (3.63), VAL367 (3.58)	
NITM4	*Justicia adhatoda* L., Syn. *Adhatoda vasica* Nees	Daucosterol	−7.6	−12.77	PHE342 (3.23), SER373 (3.72), TRP436 (3.13) ARG509 (2.94)	LEU335 (3.41), PHE338 (3.55), PHE342 (3.51), ASP364 (3.94), VAL367 (3.72), PHE374 (3.80)	
NITM5	*Ocimum tenuiflorum* L., Syn. *Ocimum sanctum* L.	Caryophyllene	−8.1	−13.77	PHE338 (2.87), GLY339 (2.88), SER373 (2.70)	LEU335 (3.00), PHE338 (3.75), PHE342 (3.89), ASP364 (3.30), VAL367 (3.62), LEU368 (3.19), PHE374 (3.72)	
NITM6	*Zingiber officinale* Roscoe	Geraniin	−8.2	−9.40	VAL362 (3.79), ASP364 (3.04), VAL367 (3.29), SER371 (3.81)	LEU368 (3.68)	
NITM7	*Curcuma longa* L.	O-Demethyl demethoxycurcumin	−8.0	−7.84	CYS336 (3.04), ASP364 (3.84)	LEU335 (3.70), PHE338 (3.42), ASP364 (3.67), VAL367 (3.55), LEU368 (3.95), PHE374 (3.85)	PHE374, TRP436
NITM8	*Syzygium aromaticum* L.	Tellimagrandin-II	−8.2	−15.68	CYS336 (2.90), PRO337 (3.52), GLY339 (3.72), GLU340 (3.37), ASN343 (3.15), ASP364 (3.73), VAL367 (3.72), SER371 (2.91), SER373 (2.93)	PHE338 (3.28), VAL367 (3.47), LEU368 (3.39)	

*Binding energy—Autodock binding score; KDeep ΔG—absolute protein ligand binding affinity calculated using KDeep tool*.

**Figure 6:**
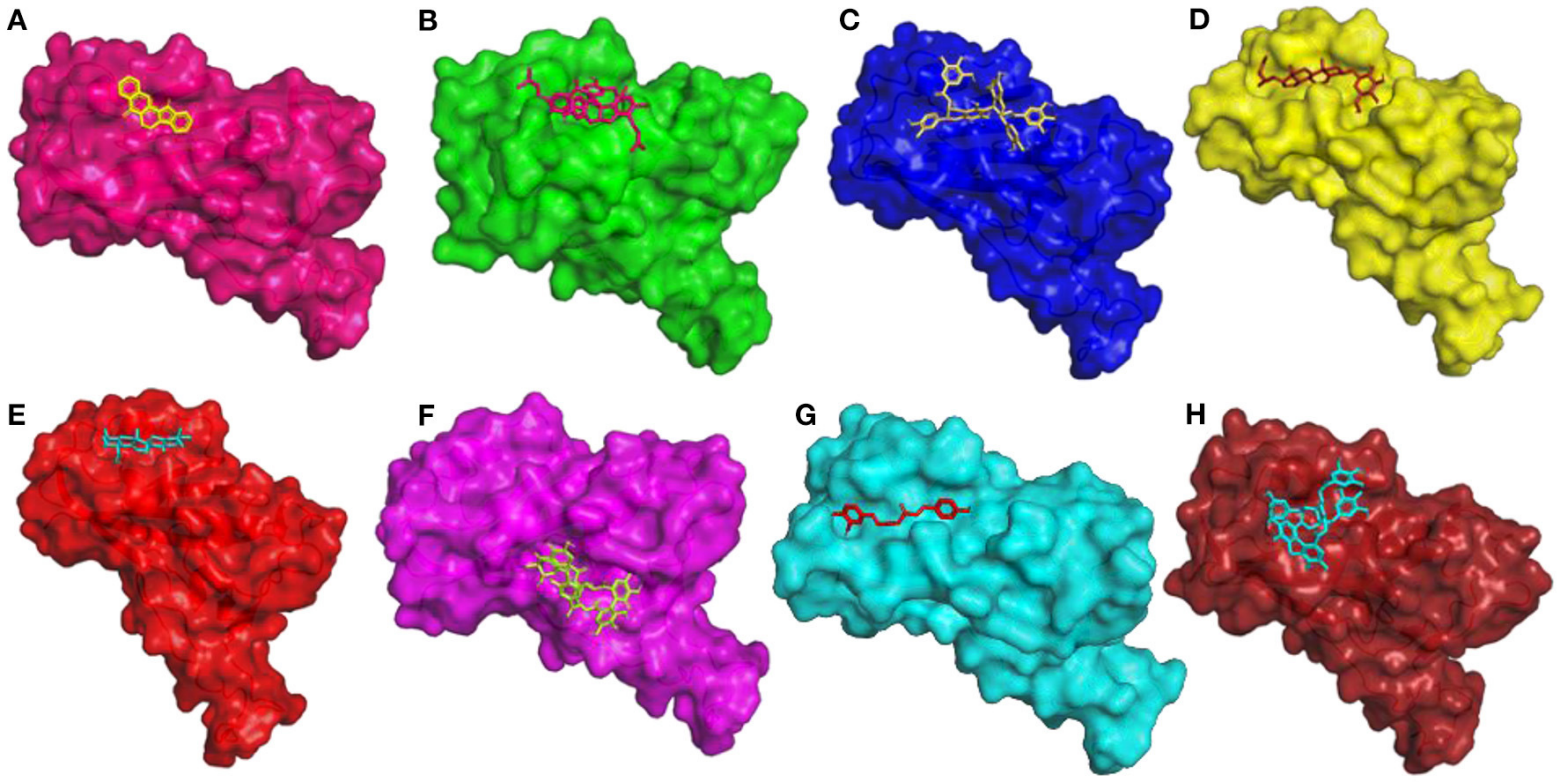
3D diagram of RBD of Spike Glycoprotein in complex with **(A)** Rutaecarpine, **(B)** Licoagrodin, **(C)** 3-O-Galloylepicatechin-(4Beta-6)-Epicatechin-3-O-Gallate, **(D)** Daucosterol, **(E)** Caryophyllene, **(F)** Geraniin, **(G)** O-Demethyldemethoxycurcumin, and **(H)** Tellimagrandin-II.

The compound Rutaecarpine from *T. indica* was found to be interacting with spike protein at SER371 and SER373 with a binding affinity of −7.9 kcal/mol (Figure 6A). Tylophorinidine showed a hydrogen bond with ASN343 (glycosylation site) and binding energy of −6.9 kcal/mol. Licoagrodin from *G. glabra* was found to interact with GLY339, ASP364, VAL367, and SER371 of RBD region with a binding affinity of −8.7 kcal/mol (Figure 6B). Further analysis also showed that Hispaglabridin-B, Licoagrone, and Licocoumarin-A from *G. glabra* to form hydrogen-bonded interactions with glycosylation site ASN343 with a binding energy of −8.2 kcal/mol, respectively. Cryptoxanthin showed hydrogen bond with ASN440, stabilized by 7 hydrophobic interactions having a binding energy of −8.4 kcal/mol (Figure 6C); while 3-O-Galloylepicatechin-(4Beta-6)-Epigallo-catechin-3-O-Gallate (−8.3 kcal/mol) showed hydrogen bonded interactions at positions: PHE338, ASN370, SER371, SER373, ASN437, and ASN440. Based on this significance it was considered for further studies. Furthermore, compounds namely, Camelliquercetiside-B (−7.8 kcal/mol), Procyanidin C1 (−7.8 kcal/mol), 3-O-Galloylepiafzelechin-(4Beta-6)-Epigallo-catechin-3-O-Gallate (−7.7 kcal/mol), Theasinensin B (−7.6 kcal/mol), Epigallocatechin-(2 Beta-7,4 Beta-8)-Epigallocatechin-3-O-Gallate (−7.5 kcal/mol), 3-O-Galloylepicatechin-(4 Beta-8)-Epicatechin-3-O-Gallate (−7.3 kcal/mol), and Theasinensin-C (−7.2 kcal/mol) from *C. sinensis* also showed hydrogen bond with residue ASN343. Compounds from *J. adhatoda* did not show any interactions with active residues, however interactions were observed with residues proximal to the active region: PHE342, SER371, and SER373. Among all the compounds studied from *J. adhatoda*, Daucosterol showed the highest binding affinity

with a score of −7.6 kcal/mol (Figure 6D). In case of *O. tenuiflorum*, Stigmastanol (−7.7 kcal/mol) showed interactions with surrounding residues of active cavity and (+)-Taxifolin (−7.0 kcal/mol) formed hydrogen bonded interaction with ASN343, while other compounds showed interactions only with other residues proximal to active residues and showed higher binding affinity. Among these compounds, Caryophyllene featured the highest binding affinity with a score of −8.1 kcal/mol (Figure 6E). Compounds Isoginkgetin (−7.6 kcal/mol), Rutin (−7.3 kcal/mol), 4′-Methoxyglabridin (−7.2 kcal/mol), Curcumin (−7.2 kcal/mol), Cubebin (−7.2 kcal/mol), Cyanin (−7.0 kcal/mol), and (Z)-1,7-bis(4-hydroxy-3-methoxy-phenyl) hept-4-en-3-one (−7.0 kcal/mol) from *Z. officinale* showed hydrogen bonded interaction with active cavity residues. The marker compounds of *Z. officinale* like Gingerenone-A and B, and Isogingerenone-B showed interactions around the active residues PHE342, SER371, and SER373, wherein Geraniin showed highest affinity with a score of −8.2 kcal/mol (Figure 6F). In case of *C. longa*, Letestuianin A (−7.2 kcal/mol) showed hydrogen bonded interaction with residue ASN343. Moreover, the marker compounds like Curcumin (−7.2 kcal/mol) and its derivatives showed interactions around the active site residues CYS336, PHE342, SER371, and SER373; wherein O-Demethyldemethoxycurcumin showed significant binding affinity with a score of −8 kcal/mol (Figure 6G). In case of *S. aromaticum* Tellimagrandin-II (−8.2 kcal/mol), Rugosin-D (−7.9 kcal/mol), Syzyginin-A (−7.8 kcal/mol), Campesterol glucoside (−7.6 kcal/mol), Sitogluside (−7.7 kcal/mol), Cirrhopetalanthrin (−7.4 kcal/mol), Tellimagrandin-I (−7.4 kcal/mol), Rugosin-E (−7.3 kcal/mol), Strictinin (−7.3 kcal/mol), Tannin (−7.1 kcal/mol), Myricetin (−7.0 kcal/mol), and Quercetin (−7.0 kcal/mol) showed hydrogen bonded interactions at active residue ASN343 and other residues. Among these compounds Tellimagrandin-II showed the highest affinity with a score of −8.2 kcal/mol (Figure 6H). The 2D interaction diagrams of all the compounds are given in Supplementary Figures 2–9.

### Molecular Dynamics Simulation of Top Docked Complexes

**Figure 7:**
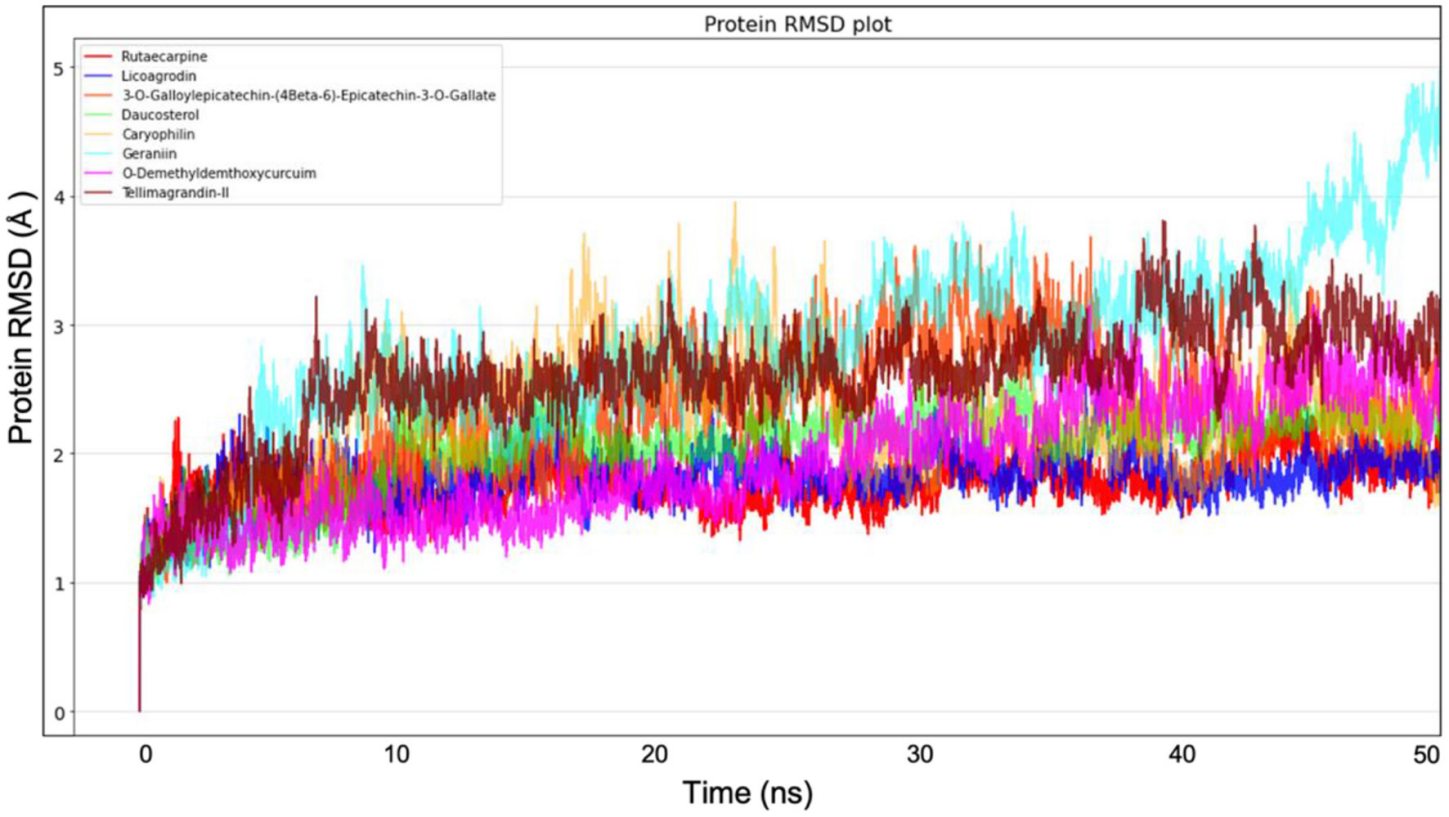
Protein Backbone RMSD plots from Molecular dynamics for all the 8 top ranking protein-ligand complexes.

**Figure 8:**
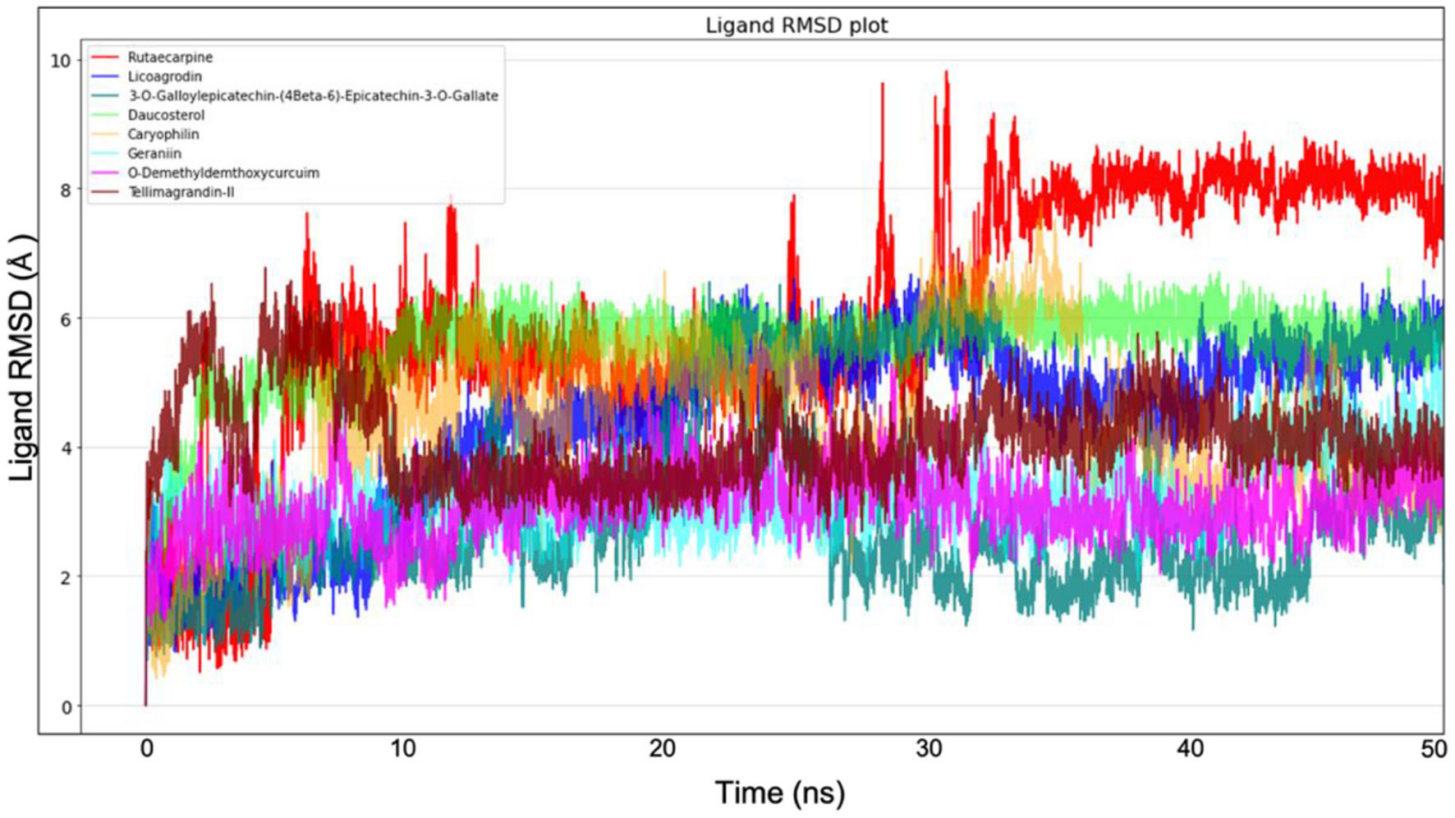
Ligand RMSD plot from Molecular dynamics for all the 8 molecules.

Molecular dynamics simulation for all the top-ranking complexes per plant was carried out with Desmond for a duration of 50 ns. The data from the trajectory was analyzed and tabulated in Supplementary Table 11. The RMSD and RMSF values of the protein backbone for all the 8 complexes were plotted and are shown in Figures 7, 8.

Based on the stability, compactness, and ligand contacts during the simulation process, Spike protein O-Demethyl-demethoxycurcumin and Spike protein Tellimagrandin-II complexes were found to be more stable and were analyzed further in detail.

#### Spike Protein O-Demethyldemethoxycurcumin Complex

**Figure 9:**
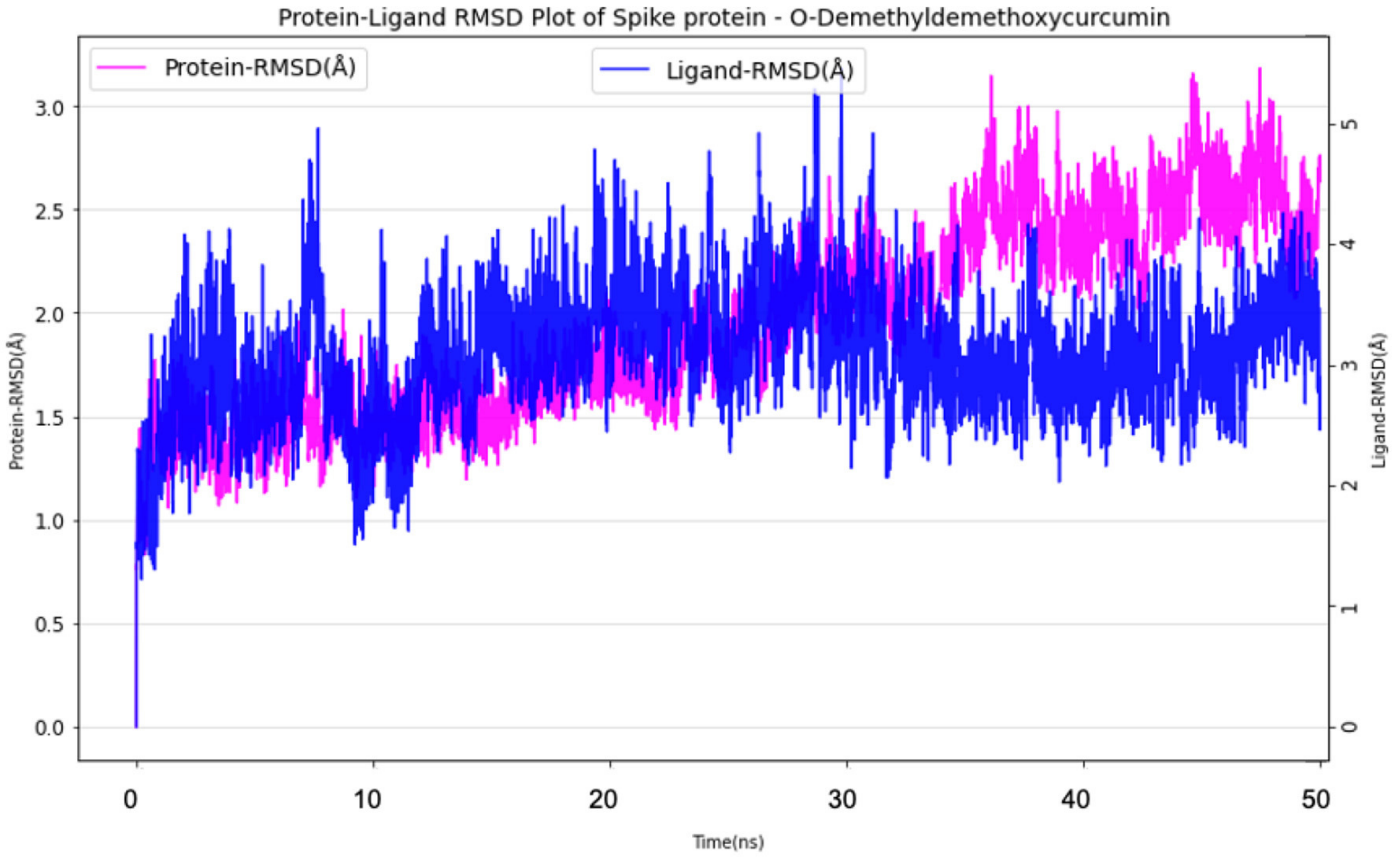
Protein-Ligand RMSD plot of Spike protein O-Demethyldemethoxycurcumin complex.

The simulation system of Spike protein O-Demethyl-demethoxycurcumin consisted of 25,902 atoms with 7,659 water molecules. To further neutralize the system, 3 Cl^−^ (7.122 mM) were added and the system was subjected to 50 ns run of production run. The RMSD plot showed a convergence at 10 ns with ~1.5 Å difference in the ligand bound state (Figure 9). The Ligand RMSD values remained within the range of 1.0–2.5 Å with average RMSD value being 1.75 Å (Figure 9). The lowest potential energy conformation was found at 21.5 ns with energy value of −84,977 kcal/mol with a binding free energy (MM-PBSA) of −12.48 kcal/mol.

The mobility of the compound in the complex during simulation with residue-wise calculations was plotted as RMSF trajectory. The analysis of the RMSF plot inferred that there was a minimum fluctuation around ~1 Å, and the trajectory to remain stable throughout the simulation with maximum deviation of~2.4 Å (Supplementary Figure 10). Further the radius of gyration (rGyr) trajectory was plotted for the entire production run, wherein the deviation was ~4.70–5.5 Å, thereby implying the higher compactness during the simulation process (Supplementary Figure 11).

**Figure 10:**
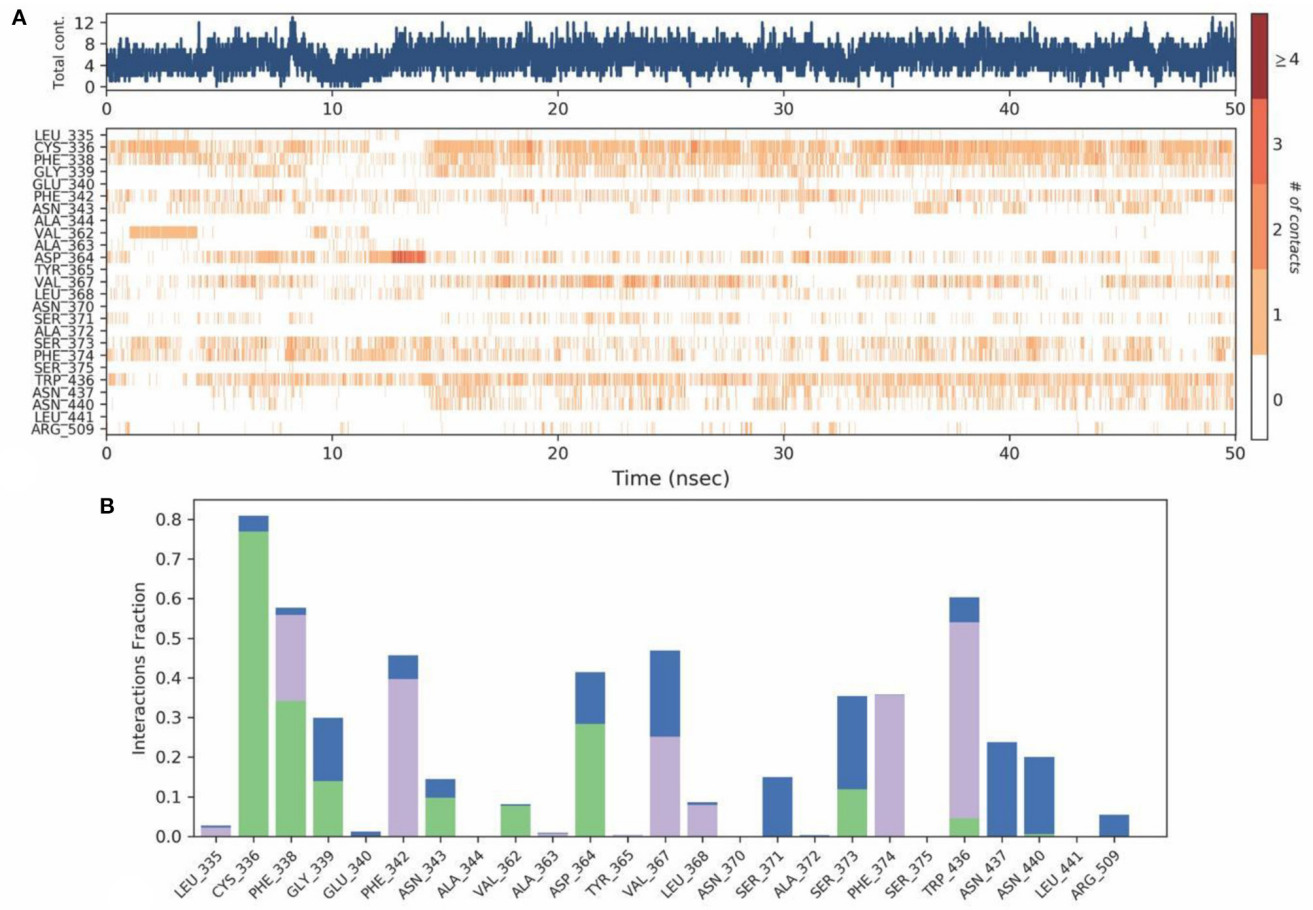
**(A)** Protein-ligand contact map for 50ns duration for Spike protein and O-Demethyl-demethoxycurcumin complex. **(B)** Protein -ligand contacts of Spike protein-O-Demethyl-demethoxycurcumin complex simulation (Blue, water bridges; Green, Hbonds; Violet, Hydrophobic).

Protein-Ligand contact analysis inferred that CYS336 to form hydrogen-bonded interactions for around 80% of the duration, followed by PHE338, which showed 60% of time to interaction by means of hydrogen bond and hydrophobic interactions. PHE342, ASP364, VAL367, and TRP436 showed around 50% of the time with interaction fraction which includes hydrophobic and water-bridge interactions (Figures 10A,B).

#### Spike Protein—Tellimagrandin-II Complex

**Figure 11:**
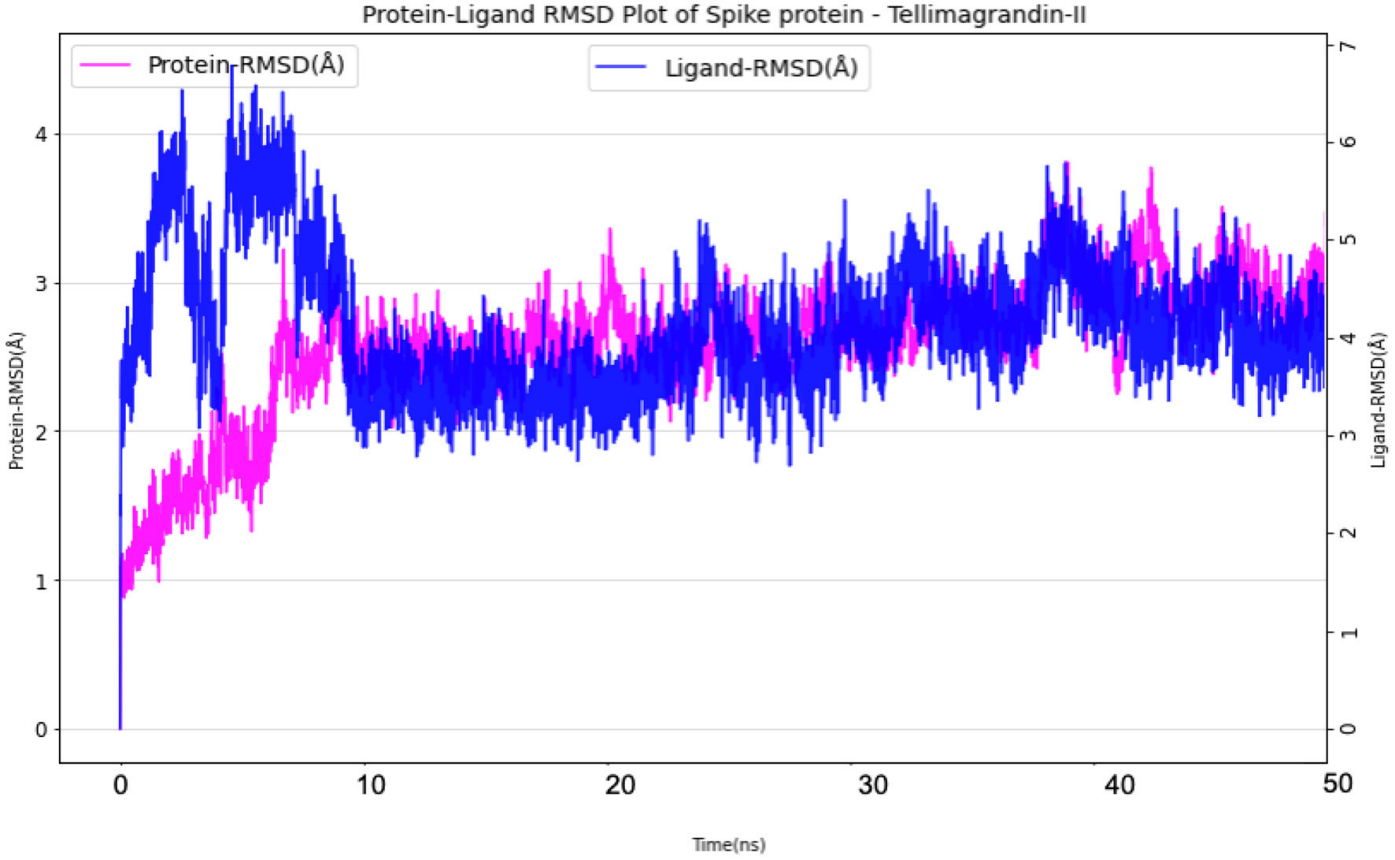
Protein-Ligand RMSD plots of Spike protein Tellimagrandin-II complex.

The simulation system of Spike protein Tellimagrandin-II complex comprised of 25,917 atoms with 7,645 water molecules. To further neutralize the system, 3 Cl^−^ (7.135 mM) were added. The system was subjected to a 50 ns run of the production run. The RMSD plot for this complex showed convergence at 10 ns with ~1 Å (admissible range) of deviation in intermolecular interactions during the entire production run (Figure 11). The Ligand RMSD values remained within the range of ~2.0–3.0 Å with an average mean value of 2.5 Å (Figure 11). The lowest potential energy conformation was found at 40.8 ns with energy value of −83,747 kcal/mol with a binding free energy (MM-PBSA) of −32.08 kcal/mol.

**Figure 12:**
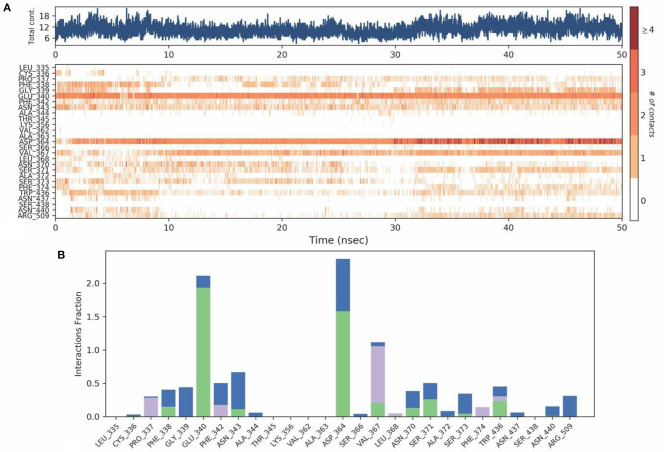
**(A)** Protein-ligand contact map for 50ns duration for Spike protein and Tellimagrandin-II complex. **(B)** Protein - ligand contacts of Spike protein Tellimagrandin-II complex simulation (Blue, water bridges; Green, Hbonds; Violet, Hydrophobic).

The mobility of the compound in the complex during simulation with residue-wise calculation was visualized as (root mean square fluctuation) RMSF plot. On further analysis, the plot inferred a minimum fluctuation in the ligand bound position ~1 Å. Moreover, the trajectory remained stable throughout the simulation with a maximal deviation of ~2.5 Å (Supplementary Figure 12). Furthermore, radius of gyration (rGyr) trajectory was also plotted, which inferred rGyr to have maintained ~5.70–6 Å, thereby implying the higher compactness of ligand (Supplementary Figure 13). Protein-Ligand contact analysis for the simulation period of 50 ns inferred that GLU340 and ASP364 to show hydrogen bonded and water-bridge interaction fraction for around 100% of the duration, with interactions at more than one position. ASN343 showed interactions around 60% of the simulation run, which mainly includes hydrogen bonds and water bridges. VAL367 showed interactions around 100% of the time includes Hydrogen bonds, Hydrophobic, and water-bridge interactions (Figures 12A,B).

## Discussion

On cumulative analysis of all the results, it could be inferred that *Tellimagrandin-II* and *O-Demethyldemethoxycurcumin*, were highly potential hits, as these compounds feature significant interactions with ACE2 binding region coupled with key glycosylation site (ASN343) of spike protein. Recent mutagenesis studies strongly suggest that the targeting ASN343 glycosylation to be the most potential inhibitory mode. Moreover, the infectivity of SARS-CoV-2 showed reduction to almost 1,200-folds when both ASN331 and ASN343 were mutated in spike protein. This shows the significance of blocking these glycosylation sites on the receptor binding domain (122). Viral glycosylation holds a major role in pathogenesis, as it mediates protein folding, shaping viral tropism, and host invasion (123). Blocking of glycosylation not only aids in preventing viral pathogenesis, but also facilitates immune recognition of the virus (124, 125). Based on the number of intermolecular interactions with active residues, glycosylation sites, and proximal residues to active site, Tellimagrandin-II with a binding energy of −8.2 kcal/mol from *S. aromaticum* may have a higher affinity toward the spike protein in comparison with all other compounds. Moreover, it also formed a stable complex, as inferred by molecular dynamics simulation. The hydrolysable tannin Tellimagrandin-II is traditionally known to possess antiviral activity; while hydrolysable tannins as a whole class are well-known antiviral agents (126). Tannins are known to inhibit various viral activities like attachment and penetration of virus and inhibition of reverse transcriptase (127). Tellimagrandin-II is the first polyphenolic ellagitannin formed from 1,2,3,4,6-pentagalloyl-glucose, and is an isomer of punicafolin or nupharin A, also known as Cornustannin 2 or Eugeniin (C_41_H_30_O_26_; Molecular Mass 93,866 g/mol). The compound is isolated from the dried flower bud of *S. aromaticum* (Clove). Earlier studies showed that ethanol extract of *S. aromaticum* to possess strong inhibition of recombinant NS2BNS3 proteases of DENV-2 and 3; while its bioactivity guided fractionation yielded eugeniin (128, 129), isobiflorin (5,7-dihydroxy-2-methylchromone-8C-β-d-glucopyranoside), and biflorin (5,7-dihydroxy-2-methylchromone-6C-β-d-glucopyranoside). Interestingly the eugeniin from *S. aromaticum* and *Geum japonicum* is found to inhibit α-glucosidase and possess significant antiviral activity against wild-type HSV-1 and HSV-2. Moreover, eugeniin also targets thymidine-kinase deficient or acyclovir as well as phosphonoacetic acid (PAA)-resistant HSV-1 at EC_50_ of 5.0 μg/ml, with CC_50_ of 69.5 μg/ml (130). Unlike nucleoside analogs, Eugeniin is reported to inhibit viral DNA polymerase and late protein syntheses in HSV-infected Vero cells, in a non-competitive manner with respect to dTTP (130). Animal studies revealed that Eugeniin at 0.3 mg/kg at oral and intraperitoneal dose retard the development of skin lesions of HSV-1-infected mice; while at 6 or 50 mg/kg it significantly prolonged the mean survival times and or reduced mortality without toxicity. However, at an oral dose of 50 mg/kg it reduced virus yields in the skin and brain of infected mice with higher bioavailability. Moreover, Eugeniin enhance the anti-HSV-1 activity of acyclovir, and interact with the polymerase near PAA-binding site (131). Eugeniin in pure form demonstrated potent inhibition of NS2BNS3 proteases of DENV-2 and 3 at IC_50_ of 94.7 nM and 7.53 μM; while moderate inhibition was found with isobiflorin and biflorin at 58.9 and 89.6 μM (132). Furthermore, the kinetic studies revealed a competitive inhibition at same binding site of both proteases; while the *K*_i_ value of eugeniin is reported as 125.2 nM for DENV2 protease, and 7.1 μM for DENV3 protease (132).

Secondly, O-Demethyldemethoxycurcumin from *C. longa* was predicted to be a promising molecule for inhibition of SARS-CoV-2 pathogenesis. It should be noted that O-Demethyldemethoxycurcumin not only confers inhibitory effects on the SARS-CoV-2 spike protein as per our prediction, but is also well-proven to be involved in Endoplasmic reticulum (ER) stress reduction (133). It is well-known that ER stress reduction is crucial in viral replication and infection, and is an essential aspect in reducing the infection level, as the complete secretory mechanism of the virus occurs in ER (134, 135). Moreover, ER stress is one of the major problems in SARS-CoV-2 infection, as the synthesis and folding of transmembrane protein loses balance and the amount of proteins entering the ER increases drastically. This loss of balance culminates in the aggregation of unfolded proteins in the ER, which in turn triggers the ER stress response that initiates to assist the organelle for homeostasis. SARS-CoV-2 activates the Unfolded protein response (UPR) and hijack the signaling pathways for its benefit to infect rapidly, hence the reduction of ER stress in the body can be a potential way of blocking SARS-CoV-2 infection (136). Curcumin and its derivatives are treated as miraculous molecules in many infectious diseases as well as in immunomodulation. The derivatives of curcumin in combination with advanced drug delivery systems may work in a multi-faceted way for the treatment and prevention of SARS-CoV-2 (88). A recent computational study showed that curcumin exhibit strong binding affinity to Spike protein of SARS-CoV-2, ACE2 receptor of host, and their complex (RBD of viral S protein and ACE2; RBD/ACE2-complex) with the binding affinity values of −7.9 kcal/mol; −7.8 kcal/mol; and −7.6 kcal/mol; – 9.1 and– 7.6 kcal/mol, respectively. Moreover, molecular dynamics simulation also substantiated the curcumin's interaction within RBD site, thereby predicts the possibility of therapeutic strategy against SARS-CoV2 (120).

The ADME and toxicity profile of O-Demethyldemethoxycurcumin and Tellimagrandin II were predicted and summarized using pKCSM (105). The Intestinal absorption of O-Demethyldemethoxycurcumin was found to be high compared to Tellimagrandin-II i.e., 76.46 and 41.54%, respectively. O-Demethyl-demethoxycurcumin was found to be CYP3A4 substrate and inhibitor of CYP1A2, CYP2C19, and CYP2C9; whereas Tellimagrandin-II was predicted as a non-inhibitor. However, both the compounds were found to be non-substrate to CYP2D6 and non-inhibitor of CYP2D6 and CYP3A4, thus shall be non-toxic. Further, both these compounds showed a negative effect in the AMES toxicity, which indicates its negligible effect on the different bacterial strains; and a negative effect on the hERG (Ether-à-go-go-Related Gene), thereby unlikely to cause arrhythmia; and do not have hepatotoxic property. The oral acute toxicity of O-Demethyl-demethoxycurcumin was predicted to be 2.23 mol/kg, whereas, for Tellimagrandin II it was 2.48 mol/kg. Similarly, the oral rat chronic toxicity of O-Demethyldemethoxycurcumin was predicted to be 2.715 log mol/kg body weights per day, whereas, for Tellimagrandin II it was 10.618 log mol/kg body weight per day. Both these compounds also scored significant KDeep ΔG (absolute binding affinity) and MM-PBSA values.

## Conclusion

The RBD of Spike protein is one of the major targets in the inhibition of SARS-CoV-2 and is the most sought-after target being worked out across the globe. Some of the key residues which are involved in the entry and infection of the SARS-CoV-2 harbors on the RBD of the spike protein. Recently, glycosylation sites are also suggested to hold a key role in viral proliferation, as inferred by mutagenesis studies. Hence, in this study, virtual screening of phytochemical inhibitors targeting RBD domain was carried out, with a key emphasis on ACE2 binding residues along with glycosylation sites. Among the compounds studied, *Tellimagrandin-II* from *S. aromaticum* and *O-Demethyl-demethoxycurcumin* from *C. longa* were found to show stable interactions with key hotspot residues (ACE2 binding) including the glycosylation site. Molecular dynamics simulation of these compounds in complex with RBD also showed higher stability due to intermolecular interactions with active residues, significant binding free energy and optimal shape complementary during the entire production run. The results from this study clearly indicates that the proposed compounds may be considered as potential candidates for the inhibition of SARS-CoV-2 infection, as these are dual-acting in terms of inhibiting ACE2 interactions, as well as targeting the glycosylation of spike protein. However, further experimental validations are warranted to infer the therapeutic efficacy.

## Data Availability Statement

The datasets presented in this study can be found in online repositories. The names of the repository/repositories and accession number(s) can be found in the article/Supplementary Material.

## Author Contributions

VU and SD performed all the computational studies which included data collection, Molecular docking and Molecular dynamics simulation, and also prepared the manuscript. The experimental design and supervision was made by VU. DC conceptualized the whole study, provided inputs and compiled the write up of traditionally used medicinal plants along with their antiviral applications available in contemporary literature, revised the manuscript, and supervised the study. HH provided initial inputs on the plants, while IS helped in the data collection. All authors contributed to the article and approved the submitted version.

## Conflict of Interest

The authors declare that the research was conducted in the absence of any commercial or financial relationships that could be construed as a potential conflict of interest.
