# Supplementary material for: Phytochemical Moieties From Indian Traditional Medicine for Targeting Dual Hotspots on SARS-CoV-2 Spike Protein: An Integrative in-silico Approach
Source: Front Med (Lausanne). 2021 May 7;8:672629. doi: 10.3389/fmed.2021.672629 (PMC8137902; doi:10.3389/fmed.2021.672629)
Supplement: Supplementary file 1 [file Data_Sheet_1.PDF]

Supplementary Data

Supplementary Tables

Table S1: Number of Compounds downloaded from each plant as per the online documented literature.

Id	Plant Name	No. of compounds documented online
NITM1	*Tylophora indica* (Burm. F.) Merrill Syn.	10
	Tylophora asthmatica (Roxb.) Wt & Arn.	
NITM2	*Glycyrrhiza glabra* L.	297
NITM3	*Camellia sinensis* L.	252
NITM4	*Justicia adhatoda* L Syn.	21
	Adhatoda vasica Nees	
NITM5	*Ocimum tenuiflorum* L Syn.	125
	Ocimum sanctum L.	
NITM6	*Zingiber officinale* Roscoe.	556
NITM7	*Curcuma longa* L.	326
NITM8	*Syzygium aromaticum* L.	365

Table S2: Compounds from *Tylophora indica* with binding score

Ligand Name	Score(Kcal/mol)
Rutaecarpine	-7.9
Hyperoside	-7
Tylophorinidine	-6.9
Stigmasterol	-6.9
Evodiamine	-6.9
Tylophorine	-6.4
Dihydroevocarpine	-6.4
(+/-)-Tylophorine	-6.3
(-)-Tylophorine	-6
Skimmianine	-5.4

Table S3: Compounds from *Glycyrrihza glabra* with binding score

Ligand name	Score (kcal/mol)
Licoagrodin	-8.7
Glyinflanin_g	-8.4
Kanzonol_d	-8.3
Liquiritic acid	-8.3
(2s,3s,4s,5r)-6-[(2s,3r,4s,5s,6s)-2-[[(3s,4ar,6ar,6bs,8as,11s,12ar,14ar,14bs)-11-carboxy-4,4,6a,6b,8a,11,14b-heptamethyl-14-oxo-2,3,4a,5,6,7,8,9,10,12,12a,14a-dodecahydro-1h-picen-3-yl]oxy]-6-carboxy-4,5-dihydroxyoxan-3-yl]oxy-3,4,5-trihydroxyoxane-2-carboxylic acid	-8.3
Dipotassium glycyrrhizinate	-8.3
Hispaglabridin_b	-8.2
Licoagrone	-8.2
Licocoumarin_a	-8.2
Liquiritigenin_7-glucoside-4'-apiosyl-(1-_2)-glucoside	-8.2
Shinflavanone	-8.2
Oleanolic acid	-8.1
18a-glycyrrhetic acid	-8.2
Glycyrrhetin	-8.2
18alpha-glycyrrhetinic acid	-8.2
(2s,3s,4s,5r,6r)-6-[(2s,3r,4s,5s,6s)-2-[[(3s,6ar,6bs,8as,11s,12ar,14bs)-11-carboxy-4,4,6a,6b,8a,11,14b-heptamethyl-14-oxo-2,3,4a,5,6,7,8,9,10,12,12a,14a-dodecahydro-1h-picen-3-yl]oxy]-6-carboxy-4,5-dihydroxyoxan-3-yl]oxy-3,4,5-trihydroxyoxane-2-carboxylic acid	-8.2
18b-glycyrrhetic acid	-8.2
(2r,3r)-3-hydroxy-2-[4-hydroxy-3-(3-methylbut-2-enyl)phenyl]-8,8-dimethyl-2,3-dihydropyrano[2,3-f]chromen-4-one	-8.1
Kanzonol_t	-8.1

(2r,4ar,6ar,6bs,10r,12ar,14bs)-10-hydroxy-2,4a,6a,6b,9,9,12a-heptamethyl-13-oxo-3,4,5,6,6a,7,8,8a,10,11,12,14b-dodecahydro-1h-picene-2-carboxylic acid	-8.1
(2s,3s,4s,5r)-6-[(2s,3r,4s,5s,6s)-2-[[(3s,6ar,6bs,8as,11s,12ar,14ar,14bs)-11-carboxy-4,4,6a,6b,8a,11,14b-heptamethyl-14-oxo-2,3,4a,5,6,7,8,9,10,12,12a,14a-dodecahydro-1h-picen-3-yl]oxy]-6-carboxy-4,5-dihydroxyoxan-3-yl]oxy-3,4,5-trihydroxyoxane-2-carboxylic acid	-8.1
(2s,3s,4s,5r,6r)-6-[(2s,4s,5s,6s)-2-[[(3s,4ar,6ar,6bs,8as,11s,12ar,14ar,14bs)-11-carboxy-4,4,6a,6b,8a,11,14b-heptamethyl-14-oxo-2,3,4a,5,6,7,8,9,10,12,12a,14a-dodecahydro-1h-picen-3-yl]oxy]-6-carboxy-4,5-dihydroxyoxan-3-yl]oxy-3,4,5-trihydroxyoxane-2-carboxylic acid	-8.1
(2s,3s,4s,5s,6r)-6-[(2s,3s,4s,5s,6s)-2-[[(3s,4ar,6ar,6bs,8as,11s,12as,14as,14bs)-11-carboxy-4,4,6a,6b,8a,11,14b-heptamethyl-14-oxo-2,3,4a,5,6,7,8,9,10,12,12a,14a-dodecahydro-1h-picen-3-yl]oxy]-6-carboxy-4,5-dihydroxyoxan-3-yl]oxy-3,4,5-trihydroxyoxane-2-carboxylic acid	-8.1
11-deoxoglycyrrhetinic acid	-8
3,2',4'-trihydroxy-6",6"-dimethyl-3'-prenylpyrano[2",3"_4,5]chalcone	-7.9
5,7-dihydroxy-3-(8-hydroxy-2,2-dimethylchromen-6-yl)chromen-4-one	-7.9
Glabridin	-7.9
Hispaglabridin_a	-7.9
Kanzonol_x	-7.9
(2s,3r,4s,5r,6r)-6-[(2r,3r,4s,5s,6s)-2-[[(3s,6ar,6bs,8as,11s,12as,14as,14bs)-11-carboxy-4,4,6a,6b,8a,11,14b-heptamethyl-14-oxo-2,3,4a,5,6,7,8,9,10,12,12a,14a-dodecahydro-1h-picen-3-yl]oxy]-6-carboxy-4,5-dihydroxyoxan-3-yl]oxy-3,4,5-trihydroxyoxane-2-carboxylic acid	-7.9
6-[2-[[(3s,4ar,6ar,6bs,8as,11s,12ar,14ar,14bs)-11-carboxy-4,4,6a,6b,8a,11,14b-heptamethyl-14-oxo-2,3,4a,5,6,7,8,9,10,12,12a,14a-dodecahydro-1h-picen-3-yl]oxy]-6-carboxy-4,5-dihydroxyoxan-3-yl]oxy-3,4,5-trihydroxyoxane-2-carboxylic acid	-7.9
Glabrol	-7.8
Glyinflanin_b	-7.8
Kanzonol_v	-7.8
Sigmoidin_b	-7.8
Glycyrrhetinic acid	-7.8
Enoxolone	-7.7
Afrormosin_7-o-(6"-malonylglucoside)	-7.7
Gancaonin_h	-7.7
Glabrone	-7.7
Isoglabrolide	-7.7
Licoflavanone_a	-7.7
Licoisoflavone_b	-7.7
Licoisoflavone-b	-7.7
Lupeol	-7.7
Pinocembroside	-7.7
(2s,3s,4s,5r,6s)-6-[[(3s,4ar,6ar,6bs,8as,11s,12ar,14ar,14bs)-11-carboxy-4,4,6a,6b,8a,11,14b-heptamethyl-14-oxo-2,3,4a,5,6,7,8,9,10,12,12a,14a-dodecahydro-1h-picen-3-yl]oxy]-5-[(2s,3r,4r)-3,4-dihydroxy-4-(hydroxymethyl)oxolan-2-yl]oxy-3,4-dihydroxyoxane-2-carboxylic acid	-7.7

6-[2-[[(6ar,6bs,8as,11s,12as,14as,14bs)-11-carboxy-4,4,6a,6b,8a,11,14b-heptamethyl-14-oxo-2,3,4a,5,6,7,8,9,10,12,12a,14a-dodecahydro-1h-picen-3-yl]oxy]-6-carboxy-4,5-dihydroxyoxan-3-yl]oxy-3,4,5-trihydroxyoxane-2-carboxylic acid	-7.7
6-[(11-carboxy-4,4,6a,6b,8a,11,14b-heptamethyl-14-oxo-2,3,4a,5,6,7,8,9,10,12,12a,14a-dodecahydro-1h-picen-3-yl)oxy]-3,4-dihydroxy-5-(3,4,5-trihydroxyoxan-2-yl)oxyoxane-2-carboxylic acid	-7.7
(2s,4as,6as,6as,6br,8ar,10s,12as,14bs)-10-hydroxy-2,4a,6a,6b,9,9,12a-heptamethyl-13-oxo-3,4,5,6,6a,7,8,8a,10,11,12,14b-dodecahydro-1h-picene-2-carboxylic acid	-7.6
Calycosin 7-o-glucoside	-7.6
3,4-didehydroglabridin	-7.6
Glyinflanin_h	-7.6
Kanzonol_c	-7.6
Licoflavone_b	-7.6
(2s,4as,6ar,6br,12as)-10-hydroxy-2,4a,6a,6b,9,9,12a-heptamethyl-13-oxo-3,4,5,6,6a,7,8,8a,10,11,12,14b-dodecahydro-1h-picene-2-carboxylic acid	-7.6
Ononin	-7.5
3-hydroxyglabrol	-7.5
Licoflavanone	-7.5
Neoliquiritin	-7.5
Licoricesaponin a3	-7.4
Glycyrrhizic acid	-7.4
(-)-phaseollinisoflavan	-7.4
Glabrocoumarin	-7.4
Glyinflanin_a	-7.4
Isoangustone_a	-7.4
Isolicoflavonol	-7.4
Kanzonol_w	-7.4
Licoagrochalcone_a	-7.4
Licoagrochalcone_b	-7.4
Licoisoflavanone	-7.4
Liquiritin	-7.4
Liquiritin_apioside	-7.4
(1r,3s,4r,7r,9s,12s,13r,17s,19r,20r,22s)-9-hydroxy-3,4,8,8,12,19,22-heptamethyl-14-oxo-23-oxahexacyclo[18.2.1.03,16.04,13.07,12.017,22]tricos-15-ene-19-carboxylic acid	-7.3
5,7-dihydroxy-3-(4-hydroxyphenyl)-6,8-bis(3-methylbut-2-enyl)chromen-4-one	-7.3
Betulinic-acid	-7.3
Glycyrrhisoflavanone	-7.3
Isoliquiritin	-7.3
Kanzonol_b	-7.3
Licoagrochalcone_c	-7.3
Licochalcone_d	-7.3
Apioglycyrrhizin	-7.3
Aescin	-7.3

Alpha,alpha'-dihydro-3,5,4'-trihydroxy-4,5'-diisopentenylstilbene	-7.2
Angustone_a	-7.2
Glabrene	-7.2
Glisoflavone	-7.2
Glycyrrhisoflavone	-7.2
Hydroxywighteone	-7.2
Kanzonol_u	-7.2
Licoagrochalcone_d	-7.2
Licocoumarone	-7.2
Liquorice	-7.2
8-prenylnaringenin	-7.1
Isoquercitrin	-7.1
3,5-dihydroxy-2-(4-hydroxyphenyl)-7-methoxy-3,4-dihydro-2h-1-benzopyran-4-one	-7.1
Neoisoliquiritin	-7.1
3',4',7-trihydroxyflavon	-7.1
Gancaonin_l	-7.1
Glabroisoflavanone_b	-7.1
Licoflavonol	-7.1
Lupiwighteone	-7.1
Shinpterocarpin	-7.1
3'-methoxyglabridin	-7
Abyssinone_ii	-7
Gancaonin_f	-7
Glyzaglabrin	-7
Isoglycycoumarin	-7
Licochalcone_c	-7
Licuroside	-7
Narcissin	-7
Quercetin	-7
Wighteone	-7
Wogonin_7-glucuronide	-7

Table S4: Compounds from *Camellia sinensis* with binding score

**Ligand name**	**Score (kcal/mol)**
Cryptoxanthin	-8.4
Zeaxanthin	-8.4
3-o-galloylepicatechin-(4beta-_6)-epicatechin-3-o-gallate	-8.3
Procyanidin-b-5-3,3'-di-o-gallate	-8.3
Lutein	-8.1
Narirutin	-8.1
3-o-galloylepigallocatechin-(4beta-_6)-epicatechin-3-o-gallate	-8
Epigallocatechin-(4beta-_8)-epicatechin-3-o-gallate	-8
3-o-galloylepicatechin-(4beta-_6)-epigallocatechin-3-o-gallate	-7.9

Violaxanthin	-7.9
Camelliquercetiside_b	-7.8
Epigallocatechin_3-o-p-coumarate	-7.8
Oolonghomobisflavan_b	-7.8
Procyanidin_c1	-7.8
Theasaponin_b1	-7.8
3-o-galloylepiafzelechin-(4beta-_6)-epigallocatechin-3-o-gallate	-7.7
Epigallocatechin_3-o-caffeate	-7.7
Epigallocatechin_3-o-cinnamate	-7.7
Egonol	-7.6
Epigallocatechin-(4beta-_8)-epigallocatechin-3-o-gallate	-7.6
Theaflagallin	-7.6
Theasinensin_b	-7.6
Epigallocatechin-(2beta-_7,4beta-_8)-epigallocatechin-3-o-gallate	-7.5
Camelliasaponin_c1	-7.4
Procyanidin_b4	-7.4
2-(3,4-dihydroxyphenyl)-5,7-dihydroxychroman-3-yl_3,4,5-trihydroxybenzoate	-7.3
3-o-galloylepicatechin-(4beta-_8)-epigallocatechin-3-o-gallate	-7.3
Betulin	-7.3
Betulinic_acid	-7.3
Epicatechin_3,5-di-o-gallate	-7.3
Gallocatechin-(4alpha-_8)-epicatechin	-7.3
Procyanidin_b3	-7.3
Strictinin	-7.3
3-o-galloylepicatechin-(4beta-_8)-epicatechin-3-o-gallate	-7.2
Camelliquercetiside_a	-7.2
Lycopene	-7.2
Theaflavin_3'-o-gallate	-7.2
Theasinensin_c	-7.2
3-o-galloylepigallocatechin-(4beta-_6)-epigallocatechin-3-o-gallate	-7.1
3-o-galloylepigallocatechin-(4beta-_8)-epigallocatechin-3-o-gallate	-7.1
Epicatechin_3-o-(3-o-methylgallate)	-7.1
(-)-epicatechin_3-o-gallate	-7.1
Epigallocatechin_3,3',-di-o-gallate	-7.1
Epigallocatechin_3,4',-di-o-gallate	-7.1
Epigallocatechin_3,5,-di-o-gallate	-7.1
Gallocatechin-gallate	-7.1
Kaempferol_3-rhamnosyl-(1-_3)(4'''-acetylrhamnosyl)(1-_6)-glucoside	-7.1
Procyanidin_b2	-7.1
Quercetin_3-(3r-glucosylrutinoside)	-7.1
Hyperoside	-7

Chlorogenic_acid	-7
Epicatechin-3-o-gallate	-7
Epicatechin_gallate	-7
Epigallocatechin_3-o-(3-o-methylgallate)	-7
Epigallocatechin-gallate	-7
Epigalocatechin_gallate	-7
Gallocatechin_3'-o-gallate	-7
Kaempferol_3-(3rha-glucosylrutinoside)	-7
Myricetin	-7
Quercetin	-7
Theasinensin_a	-7
Theasinensin_f	-7
Tr-saponin_a	-7

Table S5: Compounds from *Justicia adhatoda* with binding score

**Ligand_name**	**Score (kcal/mol)**
Daucosterol	-7.6
Genistin	-7.4
Beta-sitosterol-beta-d-glucoside	-7.3
Daidzin	-7.3
Anisotine	-7.1
Methyl_2-(methylamino)-5-(9-oxo-2,3-dihydro-1h-pyrrolo[2,1-b]quinazolin-3-yl)benzoate	-7

Table S6: Compounds from *Ocimum tenuliforum* with binding score

**Ligand_name**	**Score (kcal/mol)**
Caryophyllin	-8.1
Sitogluside	-7.7
Stigmastanol	-7.7
Ursolic acid	-7.6
Luteolin-7-o-glucuronide	-7.6
Stigmastan-3-ol	-7.6
Luteollin 5-glucoside	-7.5
Dehydroabietic acid	-7.4
Apigenin-7-o-beta-d-glucuronide	-7.4
Apigenin-7-glucuronide	-7.3
(2s,3r,4s,5s,6r)-2-[4-[(e)-2-(3-hydroxy-5-methoxyphenyl)ethenyl]phenoxy]-6-(hydroxymethyl)oxane-3,4,5-triol	-7.3
Callitrisic acid	-7.2
12-hydroxydehydroabietic acid	-7.2
Apigenin-7-o-beta-d-glucopyranoside	-7.2
Cosmosiin	-7.2
Procyanidin_b3	-7.3

Luteolin	-7.1
Rosmarinic-acid	-7.1
(+)-taxifolin	-7
Androstenedione	-7
Gob_c	-7
Grifolin	-7

Table S7: Compounds from *Zingiber offcinale* with binding score

**Ligand_name**	**Score (kcal/mol)**
Geraniin	-8.2
Isoginkgetin	-7.6
Santamarine	-7.4
Farnesiferol_a	-7.4
Rutin	-7.3
4'-methoxyglabridin	-7.2
Cubebin	-7.2
Curcumin	-7.2
Propapyriogenin A2	-7.1
Paravallarine	-7.1
9-[(2e)-3,7-dimethylocta-2,6-dienoxy]furo[3,2-g]chromen-7-one	-7.1
Isogingerenone_b	-7.1
1-(4-hydroxy-3-methoxyphenyl)-7-(4-hydroxyphenyl)hept-4-en-3-one	-7
Cyanin	-7
Delphinidin	-7
Fisetin	-7
Gingerenone-a	-7
Gingerenone-b	-7
Isogingerenone-b	-7
Myricetin	-7
Quercetin	-7
(z)-1,7-bis(4-hydroxy-3-methoxyphenyl)hept-4-en-3-one	-7

Table S8: Compounds from *Curcuma longa* with binding score

**Ligand name**	**Score (kcal/mol)**
O-demethyldemethoxycurcumin	-8
Dehydrodeguelin	-7.9
Mono-o-demethylcurcumin	-7.7
Cyqualon	-7.6
Bis-demethoxycurcumin	-7.5
Bisdemethoxycurcumin	-7.5
(1e,6e)-1-(4-hydroxy-3-methoxyphenyl)-7-(4-hydroxyphenyl)hepta-1,6-diene-3,5-dione	-7.4

Desmethoxycurcumin	-7.4
Monodemethoxycurcumin	-7.4
1,7-bis(4-hydroxyphenyl)-1-heptene-3,5-dione	-7.3
Demethoxycurcumin	-7.3
Dicinnamoylmethane	-7.3
Curcumin	-7.2
Letestuianin a	-7.2
(1e,6e)-1,7-bis(4-hydroxy-3-methoxyphenyl)hepta-1,6-diene-3,5-dione	-7.1
Letestuianin c	-7.1
(1e,4z,6e)-5-hydroxy-1,7-bis(4-hydroxyphenyl)hepta-1,4,6-trien-3-one	-7
Quercetin	-7

Table S9: Compounds from *Syzygium aromaticum* with binding score

**Ligand name**	**Score (kcal/mol)**
Tellimagrandin-ii	-8.2
Oleanolic acid	-8.1
Syzyginin b	-8.1
Rugosin-a	-8.1
Casuarictin	-8
Jervine	-8
Rugosin-d	-8
(2r,3r,4s,5s,6r)-2-[[(3s,8s,9s,10s,13r,14s,17r)-17-[(5r)-5,6-dimethylheptan-2-yl]-10,13-dimethyl-2,3,4,7,8,9,11,12,14,15,16,17-dodecahydro-1h-cyclopenta[a]phenanthren-3-yl]oxy]-6-(hydroxymethyl)oxane-3,4,5-triol	-7.9
Eugeniin	-7.9
Tellimagrandin i	-7.9
Stigmasterol glucoside	-7.8
Syzyginin-a	-7.8
[3,4,5,21,22,23-hexahydroxy-8,18-dioxo-12,13-bis[(3,4,5-trihydroxybenzoyl)oxy]-9,14,17-trioxatetracyclo[17.4.0.02,7.010,15]tricosa-1(23),2,4,6,19,21-hexaen-11-yl] 3,4,5-trihydroxybenzoate	-7.7
Pedunculagin	-7.7
Sitogluside	-7.7
Ellagitannin	-7.7
Ursolic acid	-7.6
Campesterol glucoside	-7.6
Maslinic acid	-7.5
Casuariin	-7.5
Gallotannic-acid	-7.5

1-o-galloylpedunculagin	-7.4
Cirrhopetalanthrin	-7.4
Tellimagrandin-i	-7.4
1,3-bis-(4-hydroxy-benzyl)-4-methoxy-9,10-dihydro-phenanthrene-2,7-diol	-7.3
Rugosin-e	-7.3
Strictinin	-7.3
8-(4-hydroxy-benzyl)-7-methoxy-9,10-dihydro-phenanthrene-2,5-diol	-7.2
Dehrodiisoeugenol	-7.2
Procyanidin	-7.2
Isoquercitrin	-7.1
22,23-dihydrobrassicasterol	-7.1
1-[(4-hydroxyphenyl)methyl]-4-methoxyphenanthrene-2,7-diol	-7.1
Cirrhopetalanthin	-7.1
Tannins	-7.1
Gemin-d	-7
Myricetin	-7
Quercetin	-7

Table S10 : Predicted metabolism and toxicity data of phytocompounds

Compound Name	Intestinal absorption (human)	Fraction unbound (human)	BBB permeability	CNS permeability	CYP2D6 substrate	CYP3A4 substrate	CYP1A2 inhibitior	CYP2C19 inhibitior	CYP2C9 inhibitior	CYP2D6 inhibitior	CYP3A4 inhibitior
Rutaecarpine	97.294	0.145	0.669	-1.795	No	Yes	Yes	Yes	Yes	No	Yes
Licoagrodin	100	0.325	-1.209	-2.48	No	Yes	No	No	No	No	No
3-O-Galloylepicatechin-(4Beta-6)-Epicatechin-3-O-Gallate	32.215	0.379	-3.588	-4.813	No	Yes	No	No	No	No	No
Daucosterol	79.677	0.078	-0.785	-3.021	No	Yes	No	No	No	No	No
Caryophyllin	99.931	0	-0.14	-1.157	No	Yes	No	No	No	No	No
Geraniin	84.173	0.372	-3.549	-6.083	No	No	No	No	No	No	No
O-Demethyldemethoxycurcumin	76.46	0.126	-0.947	-2.439	No	Yes	Yes	Yes	Yes	No	No
Tellimagrandin-II	41.54	0.378	-4.498	-6.005	No	No	No	No	No	No	No

Compound Name	AMES toxicity	Max. tolerated dose (human)	hERG I inhibitor	Oral Rat Acute Toxicity (LD50)	Hepatotoxicity
Rutaecarpine	Yes	0.068	No	2.431	Yes
Licoagrodin	No	0.433	No	2.478	No
3-O-Galloylepicatechin-(4Beta-6)-Epicatechin-3-O-Gallate	No	0.438	No	2.482	No
Daucosterol	No	-0.887	No	2.571	No
Caryophyllin	No	0.203	No	2.349	Yes
Geraniin	No	0.438	No	2.482	No
O-Demethyldemethoxycurcumin	No	-0.237	No	2.23	No
Tellimagrandin-II	No	0.438	No	2.482	No

Table S11: ***Molecular dynamics simulation parameters of Protein-Ligand Complexes*.**

Compound	Total Atoms	Water molecules	Ions	Ion Conc	Ligand RMSD(Å) Mean	RGyrR Range(Å)	Total Energy (kcal/mol)	Std Dev of energy
**Rutaecarpine**	25909	7663	3 Cl-	7.118mM	1.77	3.60 - 3.70	-68461.252	107.743
**Licoagrodin**	25840	7619	3 Cl-	7.159mM	1.91	5 - 5.5	-68198.100	109.112
**3-O-Galloylepicatechin-(4Beta-6)-Epicatechin-3-O-Gallate**	25882	7633	3 Cl-	7.146mM	2.59	6.0 – 6.75	-66599.11	110.906
**Daucosterol**	25924	7646	3 Cl-	7.134mM	2.02	6.4 - 7	-68222.249	107.636
**Caryophyllene**	25853	7629	3 Cl-	7.150MM	2.24	4.32 - 4.48	-68090.000	105.315
**Geraniin**	25874	7631	3 Cl-	7.147mM	2.85	5.4 - 5.85	-68288.192	107.665
**O-Demethyldemethoxycurcumin**	25902	7659	3 Cl-	7.122mM	1.94	4.5 - 5.5	-68482.892	108.417
**Tellimagrandin-II**	25917	7645	3 Cl-	7.135mM	2.58	5.70-6.0	-66784.128	230.594

**Ligand RMSD- Ligand (Root mean square deviation), RGyR- Radius of Gyration; Std Dev- Standard deviation of energy;*

Supplementary Figures

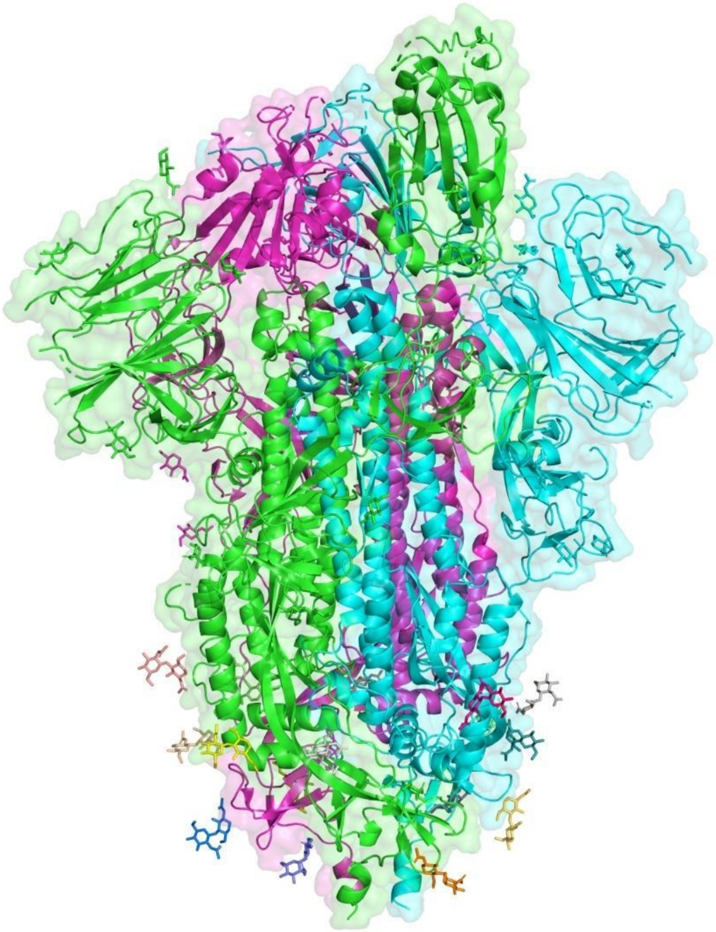

Figure S1: Homo-trimer structure of Spike glycoprotein of SARS-CoV-2 (PDB id: 6VSB) and differently coloured chains indicate the individual monomer.

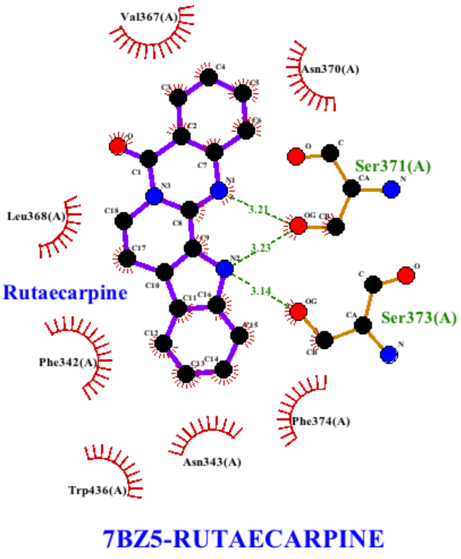

Figure S2: 2D interaction diagram of RBD of Spike Glycoprotein with Rutaecarpine

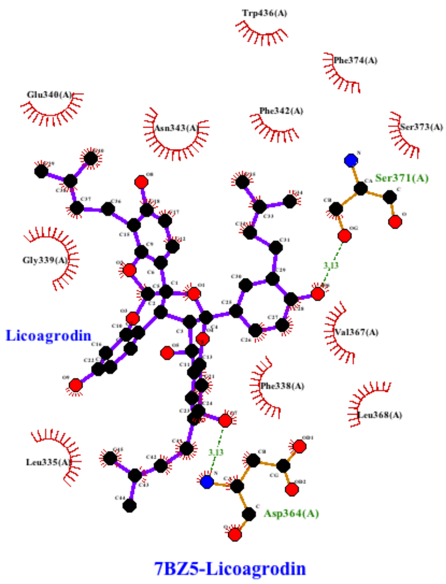

Figure S3: 2D interaction diagram of RBD of Spike Glycoprotein with Licoagrodin

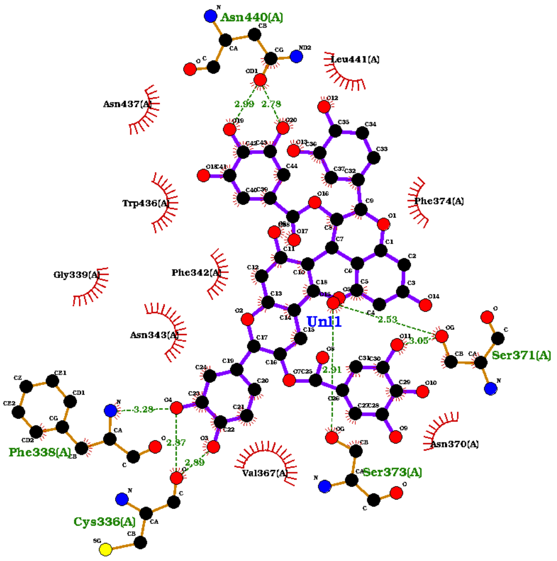

Figure S4: 2D interaction diagram of RBD of Spike Glycoprotein with 3-O-Galloylepicatechin-(4Beta-6)-Epigallocatechin-3-O-Gallate

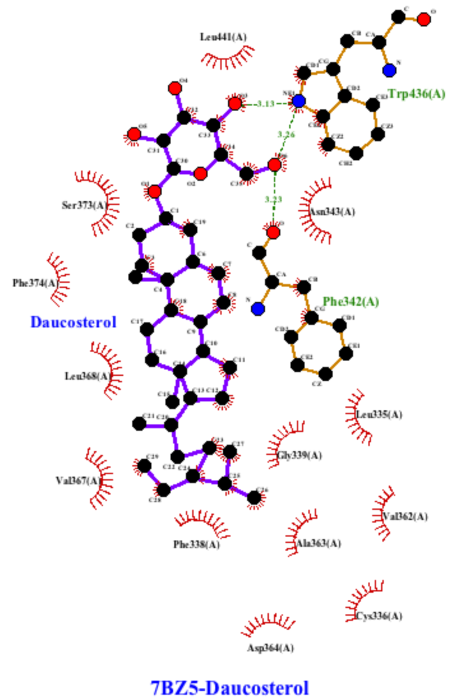

Figure S5: 2D and 3D interaction diagram of RBD of Spike Glycoprotein with Daucosterol

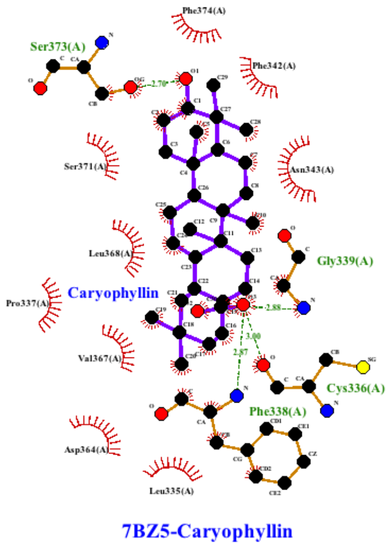

Figure S6: 2D interaction diagram of RBD of Spike Glycoprotein with Caryophyllin

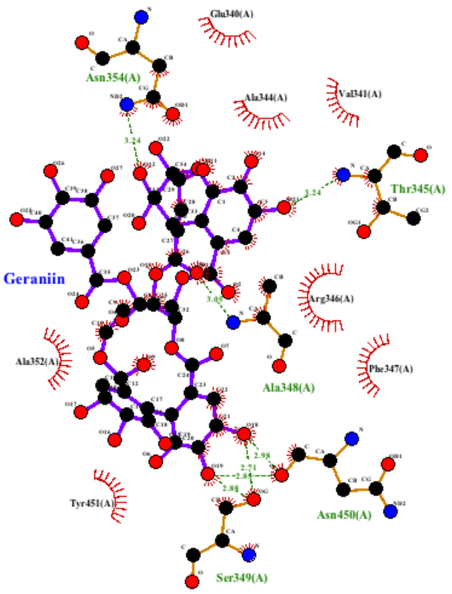

7bz5-Geraniin

Figure S7: 2D interaction diagram of RBD of Spike Glycoprotein with Geraniin

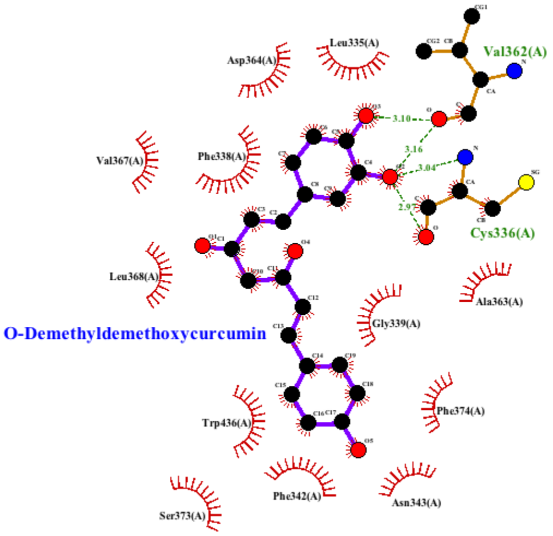

7BZ5 - O-Demethyldemethoxycurcumin

Figure S8: 2D interaction diagram of RBD of Spike Glycoprotein with O-Demethyldemethoxycurcumin.

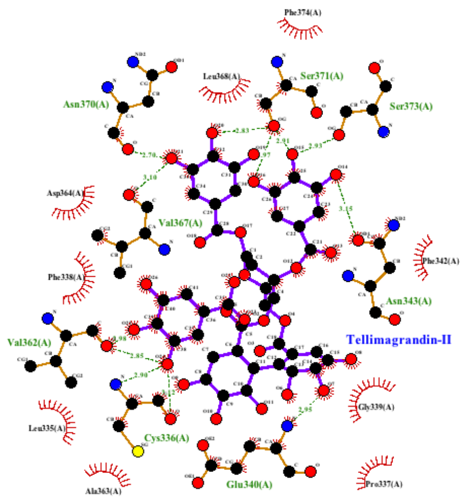

7BZ5-Tellimagrandin-II

Figure S9: 2D and 3D interaction diagram of RBD of Spike Glycoprotein with Tellimagrandin-II

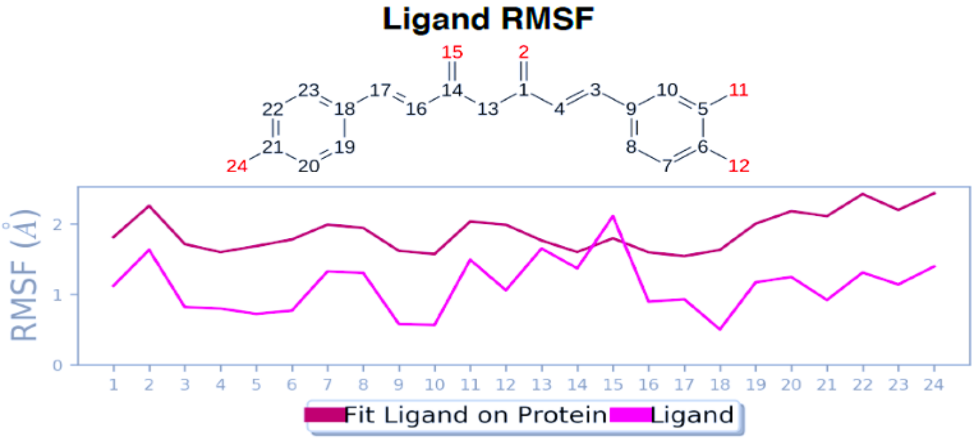

Figure S10: *Ligand RMSF plot for O-Demethyldemethoxycurcumin during Simulation of 50 ns*.

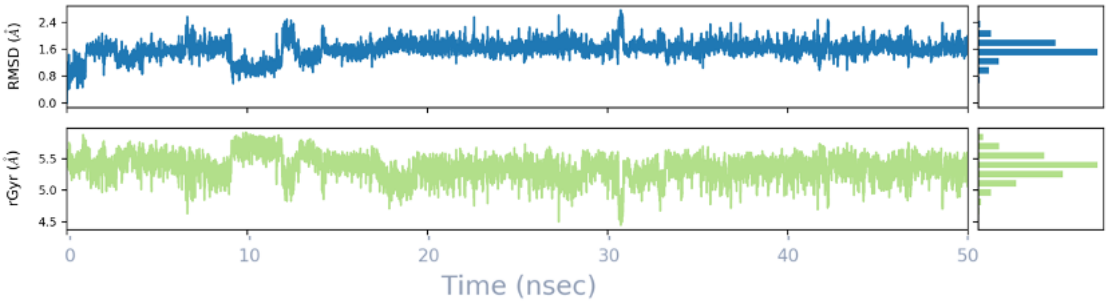

Figure S11: *Ligand RMSD and Radius of Gyration plot of O-Demethyldemethoxycurcumin.*

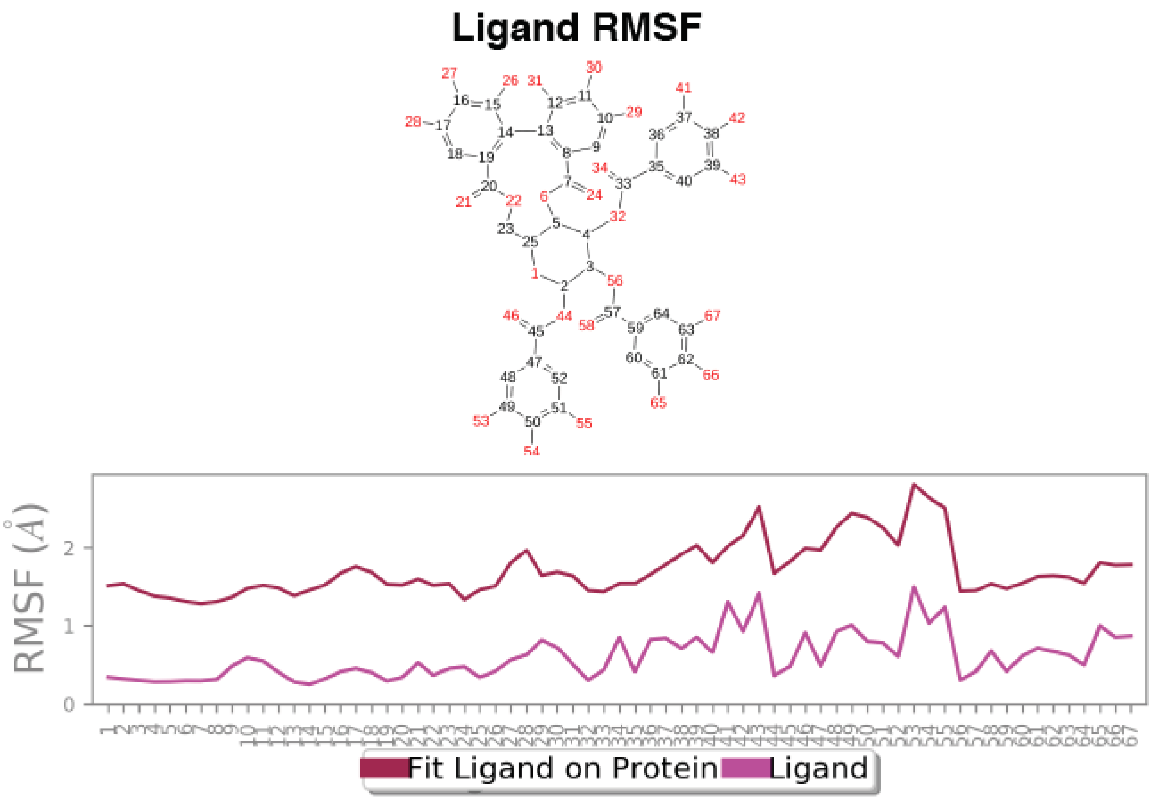

Figure S12: *Ligand RMSF plot for Tellimagrandin-II during Simulation of 50 ns*.

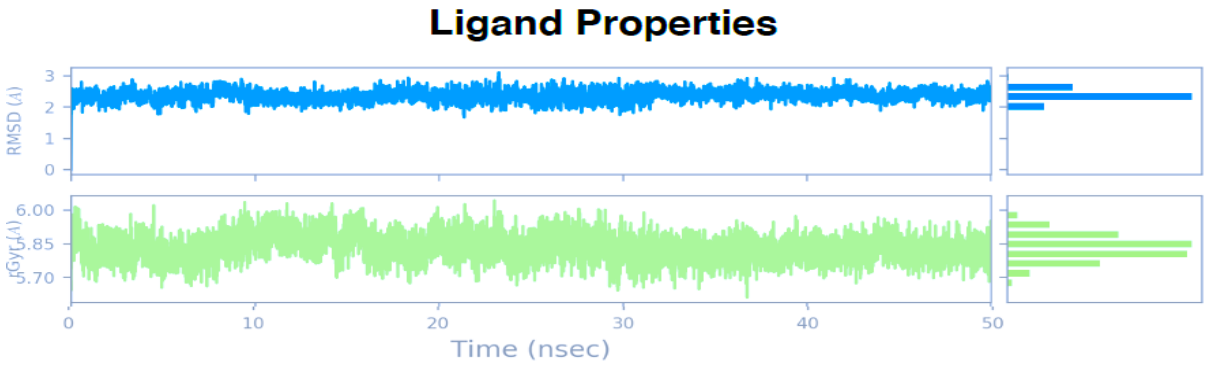

Figure S13: *Ligand RMSD and Radius of Gyration plot of Tellimagrandin-II*
